# Ultrasound Activated Piezoelectric Dural Patches to Drive Endogenous Neural Stem Cell–Mediated Repair Traumatic Brain Injury

**DOI:** 10.1002/advs.202524326

**Published:** 2026-03-05

**Authors:** Pengbo Zhou, Qingyuan Wu, Yang Wu, Runzhe Huang, Wei Li, Hanjie Niu, Hongtao Sun, Huiyu Liu

**Affiliations:** ^1^ The First School of Clinical Medical Lanzhou University Lanzhou Gansu China; ^2^ Centre for Neurological Diseases Research Characteristic Medical Center of People's Armed Police Forces Tianjin China; ^3^ Department of Chemistry Key Laboratory of Bioorganic Phosphorus Chemistry & Chemical Biology Tsinghua University Beijing China; ^4^ Beijing Advanced Innovation Center for Soft Matter Science and Engineering State Key Laboratory of Organic‐Inorganic Composites Beijing Laboratory of Biomedical Materials Bionanomaterials & Translational Engineering Laboratory Beijing Key Laboratory of Bioprocess Beijing University of Chemical Technology Beijing P. R. China; ^5^ Department of Neurosurgery The Second Hospital of Hebei Medical University Shijiazhuang China

**Keywords:** neural stem cells, piezoelectricity, Poly(L‑lactic acid), traumatic brain injury, ultrasound

## Abstract

Endogenous neuronal differentiation of neural stem cells (NSCs) is a promising route to restore function after traumatic brain injury (TBI), but direct transplantation of exogenous NSCs faces practical and immunological barriers and yields limited neuronal maturation. Here, a clinically relevant strategy is reported that converts a dura mater into an active piezoelectric patch to noninvasively drive endogenous NSC neurogenesis. Electrospun poly(L‑lactic acid) (PLLA) patches were subjected to surface confinement crystallization on metal substrates, producing a metastable α′ crystal structure and markedly enhanced piezoelectric output. Under low‑intensity transcranial ultrasound, the treated patch generates reproducible pulsed electrical signals that remodel the local injury microenvironment. In vitro and in vivo assays show that ultrasound‑activated patches increase neuronal lineage differentiation (neurons/astrocytes ratio increased ∼9.6‑fold at 14 days) and promote greater neuronal maturation, while concomitantly modulating the immune milieu. In a rat TBI model, daily 2‑min ultrasound stimulation delivered via the patch substantially accelerated tissue repair and improved behavioral and cognitive outcomes compared with untreated controls. This work demonstrates a simple, scalable modification of clinical artificial dura mater to produce a soft, biodegradable piezoelectric implant capable of remote, noninvasive electrical modulation of endogenous NSCs, with broad implications for neural regeneration and potential clinical translation.

## Graphical abstract

This study presents a wireless, non‐invasive strategy for neural repair by developing a biodegradable piezoelectric dural patch that, under transcranial ultrasound, generates localized electrical fields to drive endogenous neural stem cells toward neuronal differentiation and functional integration. Through combined material innovation, multimodal biological validation, and mechanistic investigation, we demonstrate that ultrasound‐activated piezoelectric stimulation effectively promotes neurogenesis, modulates neuroinflammation, and significantly enhances structural and functional recovery after traumatic brain injury.

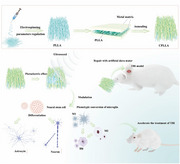

## 1 Introduction

Traumatic brain injury (TBI) represents a major global challenge in neuroscience and public health, imposing substantial mortality, long‐term disability and socioeconomic burden [1]. Primary TBI disrupts the blood‐brain barrier, causes loss of neurons and glia, and triggers local inflammation and secondary neurodegeneration [2, 3]. Importantly, the brain's principal neurogenic niches, including the dentate gyrus of the hippocampus and the subventricular zone, are activated after injury, indicating that endogenous neurogenesis is a key contributor to functional recovery [4, 5, 6]. Therefore, strategies that effectively promote neuronal regeneration and reconstruct a permissive repair microenvironment are central to TBI therapy [1].

Neural stem cells (NSCs) derived from the central nervous system possess self‑renewal capacity and multipotency, enabling differentiation into neurons and the major glial cell types [7]. The capacity to promote the differentiation of endogenous NSCs into functional neurons, thereby replacing damaged cells and reconstructing neural circuits, offers novel therapeutic avenues for TBI [8]. However, within the injured microenvironment, the majority of NSCs differentiate into astrocytes, thereby contributing to scar formation and significantly impairing neural network function [9, 10]. Although transplantation of exogenous NSCs can augment neurogenesis and improve functional outcomes in TBI models, clinical translation remains limited by poor graft survival, procedural complexity and host immune responses [11, 12, 13, 14]. Endogenous NSCs offer an attractive alternative, but their activation, differentiation pace and lineage commitment are often insufficient to restore complex circuitry after injury [15]. Electrical stimulation is an established modality to enhance neurogenesis and neuronal differentiation through modulation of membrane receptors, ion channels and intracellular signaling [16, 17, 18]. However, conventional electrical stimulation relies on implanted electrodes and wired power sources, which add surgical risk, inflammation and infection, and make precise stimulation of grafted or endogenous single cells challenging [19]. Recently, non‑invasive remote stimulation approaches have emerged; for example, piezoelectric materials that generate electrical signals under ultrasound have been applied to modulate peripheral nerve and spinal cord repair [17, 20]. Many high‑performance piezoelectric ceramics (e.g., barium titanate) are attractive for their large piezoelectric coefficients, but their mechanical mismatch with soft brain tissue and poor long‑term integration can provoke chronic fibrosis and loss of function following implantation [21, 22]. Thus, there is an unmet need for soft, biocompatible and biodegradable piezoelectric materials capable of delivering efficacious electrical cues to accelerate endogenous NSC differentiation while minimizing foreign‑body responses.

Poly(L‑lactic acid) (PLLA) is an FDA‑approved, biodegradable polymer that exhibits intrinsic piezoelectricity arising from the oriented ester carbonyl dipoles along its helical chains, making it a promising candidate for implantable piezoelectric devices [23]. Nevertheless, the native piezoelectric output of PLLA is modest and must be enhanced to generate meaningful electrical stimulation under clinically safe ultrasound amplitudes. Crucially, PLLA's piezoelectricity depends on its crystalline packing: in the thermodynamically stable α polymorph, carbonyl dipoles are partially cancelled by antiparallel packing, whereas ordering of carbonyl dipoles along the chain axis can produce a net polarization and substantially increase the piezoelectric response [24, 25].

In this study, a controlled confinement crystallization strategy is reported to modulate the crystal structure of PLLA patches, leading to amplified ultrasound‐driven electrical output. Through guided thermal recrystallization on metal substrates, PLLA is biased toward polar crystalline arrangements, thereby enhancing its piezoelectric transduction under pulsed ultrasound. Furthermore, the treated patches are shown to accelerate the differentiation and maturation of NSC in vitro. When configured as flexible patches and applied to the injured cortex in a rat TBI model, these patches generate ultrasound‐synchronized pulsed electrical stimulation that promotes endogenous NSC differentiation into functional neurons, supports tissue repair, and significantly improves behavioral and cognitive outcomes.

## 2 Results and Discussion

### 2.1 Synthesis and Characterization of PLLA and CPLLA

**FIGURE 1:**
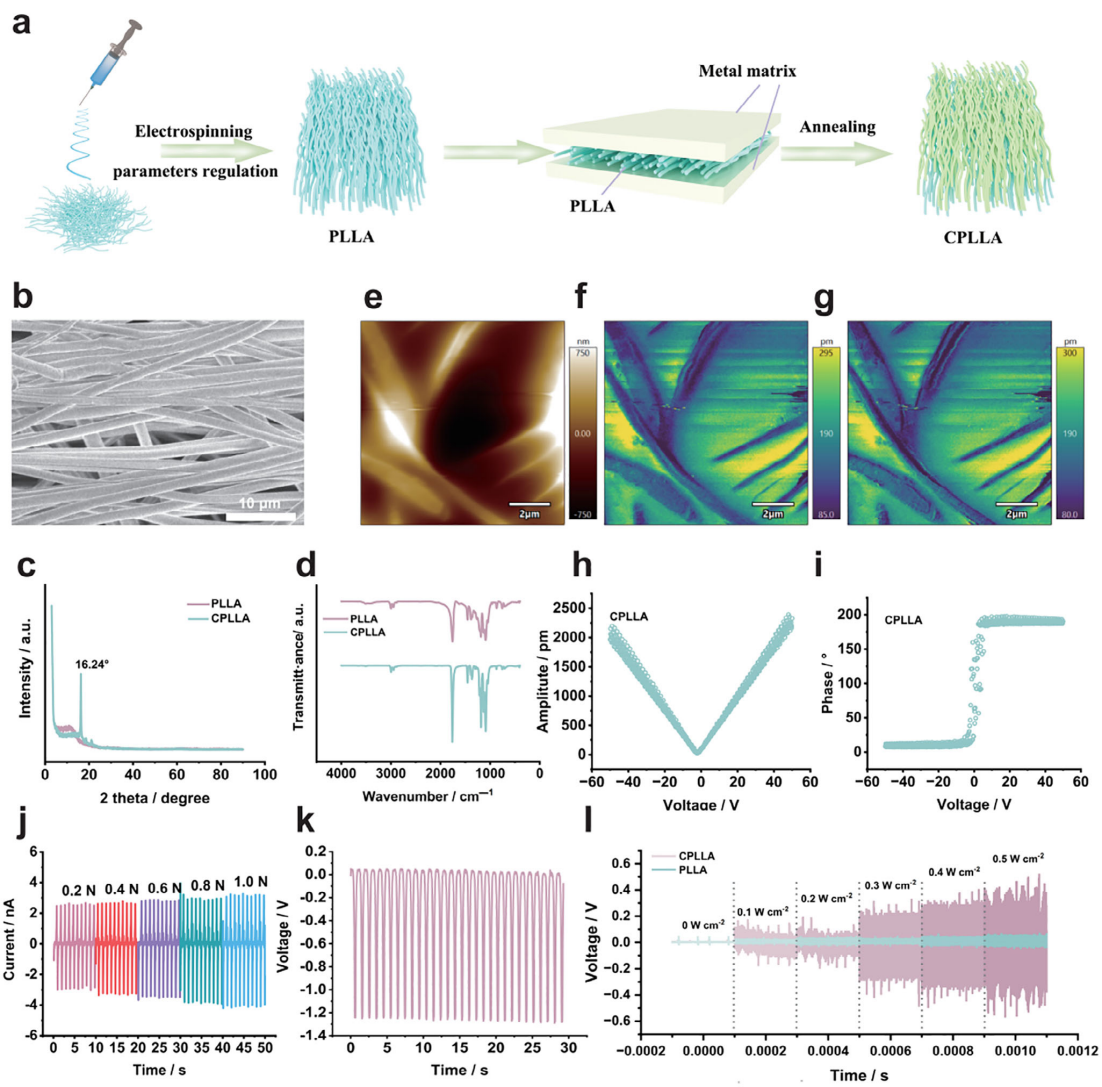
(a) The schematic illustration of the synthesis for CPLLA. (b) SEM image of CPLLA. (c) XRD patterns of CPLLA and PLLA. (d) FTIR spectra of CPLLA and PLLA. (e) surface topography, (f) amplitude plot, (g) phase diagram, (h) amplitude curve, and (i) phase curve of CPLLA. (j) Current output of CPLLA under different forces. (k) The voltage output of CPLLA under the mechanical pressure of 1 N. (i) Voltage output of CPLLA and PLLA under different ultrasound intensity.

Electrospun PLLA fiber patch were fabricated (Figure 1a). Under identical processing parameters, isopropanol as a solvent produced markedly more uniform fibres than dichloromethane (Figures S1 and S2). Increasing the collector rotation from 300 to 1600 rpm yielded a highly aligned patch with an average fibre diameter of ∼1.4 µm as observed by scanning electron microscope (SEM) (Figure S3). To enhance crystallinity, the patches were thermally annealed. Conventional thermal treatment yielded a characteristic diffraction peak at 16.7°, which is indicative of the thermodynamically stable α polymorph (Figure S4). This resulting crystal structure, however, was incompatible with the targeted microstructural requirements.

During the crystallization of PLLA, ester groups on adjacent molecular chains attract each other via dipole–dipole interactions, with the carbonyl group (C═O) of one chain facing the oxygen atom of another. The molecular chains adopt a specific 10/3‐helix conformation and pack closely to form the stable α crystal form. To modulate the components that may raise safety concerns, we sought a method to guide chain arrangement. Given that the carbonyl groups and oxygen atoms in PLLA chains possess lone‐pair electrons capable of coordinating with vacant orbitals of transition metal atoms, we hypothesized whether such interactions could promote ordered alignment of PLLA chains and induce the formation of a specific crystal polymorph [26]. In this study, various common metal sheets, used as‐received without intentional removal of surface oxide layers, were selected as substrates to facilitate pre‐alignment and immobilization of PLLA chains. After thermal treatment for 5 h, XRD analysis was performed. The patterns indicated that the metal substrates influenced the crystallization behavior of PLLA, including the peak at 16.7° disappeared and was replaced by a broad peak around 13.2°. Among them, aluminum substrates induced the most noticeable peak enhancement of the treated PLLA, suggesting the possible formation of a short‐range ordered mesophase within the material (Figure S5). On the basis of these results, we used aluminum substrates and extended the thermal anneal to 10 h to fabricate a crystalized PLLA (CPLLA) patch. The SEM image also shows the diameter of CPLLA as around 1.4 µm (Figure 1b). The XRD patterns of CPLLA and untreated PLLA then diverged markedly (Figure 1c). CPLLA displayed a sharp peak at 16.3°, consistent with formation of the metastable α′ polymorph. The α′ phase is commonly observed under constrained crystallization or at lower crystallization temperatures. Compared with the dense, ordered orthorhombic α phase, α′ exhibits looser chain packing, a slight lattice expansion, weakened long‐range order, and greater lattice distortion. Fourier Transform Infrared Spectroscopy (FTIR) spectra also revealed differences between PLLA and CPLLA. Both samples show the carbonyl stretch near ∼1750 cm^−1^, but CPLLA exhibits a pronounced change in peak shape. The C–O–C main‐chain vibrations (1180–1080 cm^−1^) show small shifts and intensity changes, indicating adjustments in backbone conformation and interchain interactions (Figure 1d) [27].

### 2.2 Enhanced Piezoelectric Properties and Functional Output

Piezoresponse force microscopy (PFM) characterization confirmed a substantial enhancement in the piezoelectric response of the CPLLA compared to its conventionally treated counterpart. The effective piezoelectric coefficient, derived from PFM amplitude curves, increased markedly from approximately 12 pm V^−1^ (Figure S6) for pristine PLLA to about 44 pm V^−1^ for CPLLA (Figure 1e–i), indicating a nearly threefold improvement. This significant augmentation can be attributed to the optimized polar crystalline orientation and higher degree of structural order induced by the confinement strategy. In addition, the experimental results indicate that CPLLA demonstrates an enhanced piezoelectric response at lower bias voltages, yielding a higher effective piezoelectric coefficient compared to high‐bias states (Figure S7). The experiment indicating that fibrous membranes obtained via confined crystallization in this work exhibit superior piezoelectric performance compared to most reported values (Table S1). Furthermore, under periodic mechanical stimulation, CPLLA consistently generated well‐defined electrical output pulses, reaching ∼1.3 V in voltage and ∼3.05 nA in current (Figure 1j,k), showing a more stable electrical output than PLLA (Figure S8). These output levels not only underscore the material's enhanced piezoelectric activity but also demonstrate its potential to produce electrical signals of biologically relevant magnitudes, supporting its suitability for application in mediating piezoelectricity‐driven cellular responses in a therapeutic context. In this study, the piezoelectric performance of CPLLA patch was investigated, yielding an open‐circuit voltage of 1.2 V and a short‐circuit current piezoelectric output of 5 nA upon application of 1.0 N pressure. Similarly, the piezoelectric response of the sample was characterized by capturing the output voltage signal generated upon ultrasonic excitation using an oscilloscope (Figure 1i). A distinct, periodic signal was observed, with a peak‐to‐peak amplitude confirming the effective electromechanical conversion of the material. Further investigation was conducted to evaluate the dependence of the piezoelectric output signal on the ultrasonic excitation power. The output voltage for both materials exhibits a monotonic increase with rising ultrasonic power, indicating a linear relationship between the input mechanical energy and the electrical response. However, CPLLA demonstrates a significantly steeper slope in its voltage response. Specifically, at an ultrasonic power of 0.3 W cm^−2^, CPLLA generates a substantial output voltage of 0.2 V, whereas PLLA yields a negligible signal of only 0.029 V under identical conditions. This corresponds to an output nearly 7 times higher for CPLLA compared to PLLA, providing compelling evidence of CPLLA's superior electromechanical conversion efficiency and piezoelectric sensitivity. This intensity exceeds the electrical activation threshold of conventional membrane receptors [28]. It is evident from extant literature that electric field intensities within the range of 0.1–1 mV mm^−1^ have been demonstrated to induce electrophysiological responses [29]. It has been demonstrated that fields of 5–20 mV mm^−1^ effectively activate electrical activity in primate neurons, including human neurons [30]. In addition, fields of 20–50 mV mm^−1^ effectively activate electrical activity in rodent neurons [31, 32]. Consequently, the electric field strength achieved in this study is sufficient to exert electrical stimulation on NSCs. The piezoelectric coefficient was enhanced by calcination, building upon conventional L‐polylactic acid. In addition, the patch displays a thickness of approximately 2 µm and exhibits excellent flexibility, thus facilitating effective mimicry of the dura mater biological layer. This facilitates comprehensive adhesion to brain tissue while providing stem cells with a sustained, stable electrical stimulation environment.

### 2.3 Theoretical Insights Into the Substrate‐Guided Crystallization Mechanism

**FIGURE 2:**
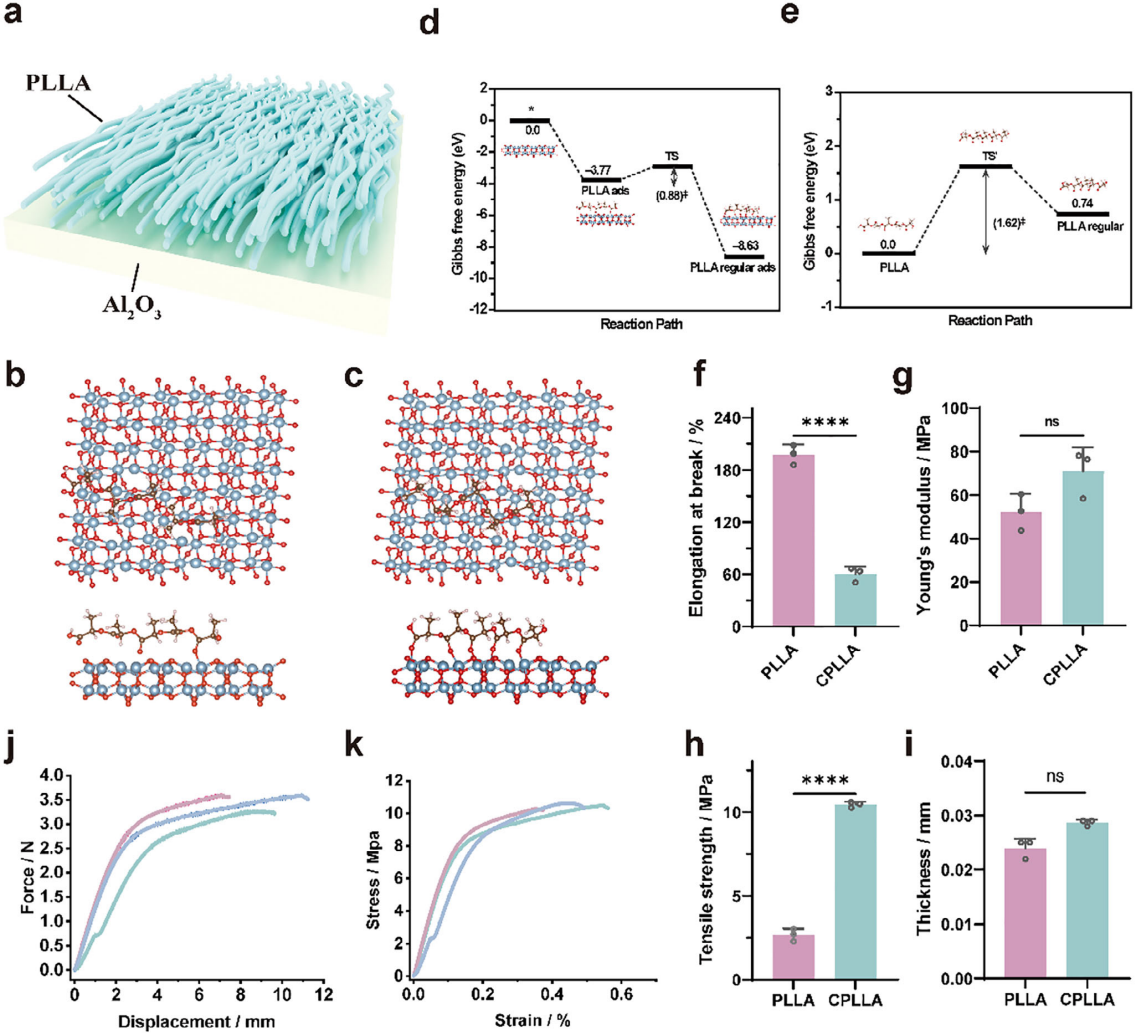
(a) Schematic of confinement crystallization synthesis for CPLLA. (b) AIMD simulation snapshot of the PLLA/Al_2_O_3_ system. (c) DFT‐optimized adsorption configuration of PLLA on Al_2_O_3_ (regular adsorption). (d,e) Gibbs free energy plots for PLLA adsorption with and without Al_2_O_3_. (g) Elongation at break, (h) Young's modulus, (i) tensile strength, (j) displacement‐force curve, and (k) strain–stress curve.

To elucidate the atomic‐scale mechanism behind the aluminum substrate‐mediated crystal modulation of PLLA, ab initio molecular dynamics (AIMD) and density functional theory (DFT) simulations were employed. Given that the native oxide layer was not intentionally removed from the aluminum substrate in the experiment, an Al_2_O_3_ (110) surface model, which represents a dominant facet, was constructed to investigate its influence on PLLA crystallization behavior (Figure 2a–c). DFT calculations revealed an adsorption energy of –3.77 eV for a single PLLA chain on the Al_2_O_3_ (110) surface. Upon adopting a regularly aligned conformation via carbonyl rotation, the adsorption energy significantly strengthened to –8.63 eV, indicating a more stable interfacial interaction between the ordered PLLA structure and the alumina surface (Figure 2d). Further DFT analysis evaluated the energy barrier for the conformational transition of PLLA on the Al_2_O_3_ (110) surface at the experimental crystallization temperature of 105°C. The computed barrier was 0.88 eV, a moderate range that can be overcome under the applied thermal conditions. In contrast, the transition of an isolated PLLA chain into an ordered configuration in the absence of the substrate required a substantially higher barrier of 1.61 eV and was endothermic by 0.72 eV, indicating both kinetic and thermodynamic impediments to spontaneous ordering without interfacial guidance (Figure 2e). AIMD simulations were subsequently performed to dynamically trace the adsorption and alignment process of PLLA on the Al_2_O_3_ (110) surface (Movie S1). As shown in Figure S9, the temperature and potential energy of the PLLA/Al_2_O_3_ (110) system rapidly equilibrated and maintained stable oscillations, confirming the robustness of the simulation. A representative snapshot of the equilibrated structure (Figure 2f), together with the dynamic trajectory, visually demonstrates that PLLA chains preferentially adopt a well‐aligned arrangement on the alumina surface. This ordered adsorption, facilitated by strong interfacial interactions, is consistent with the experimentally observed facilitation of crystallization into the targeted polymorphic form. Remarkably, after the crystallization treatment, CPLLA still exhibits excellent mechanical properties, including high tensile strength, Young's modulus and elongation at break (Figure 2g–i).

### 2.4 The Piezoelectric Properties and Biocompatibility of PLLA and CPLLA

**FIGURE 3:**
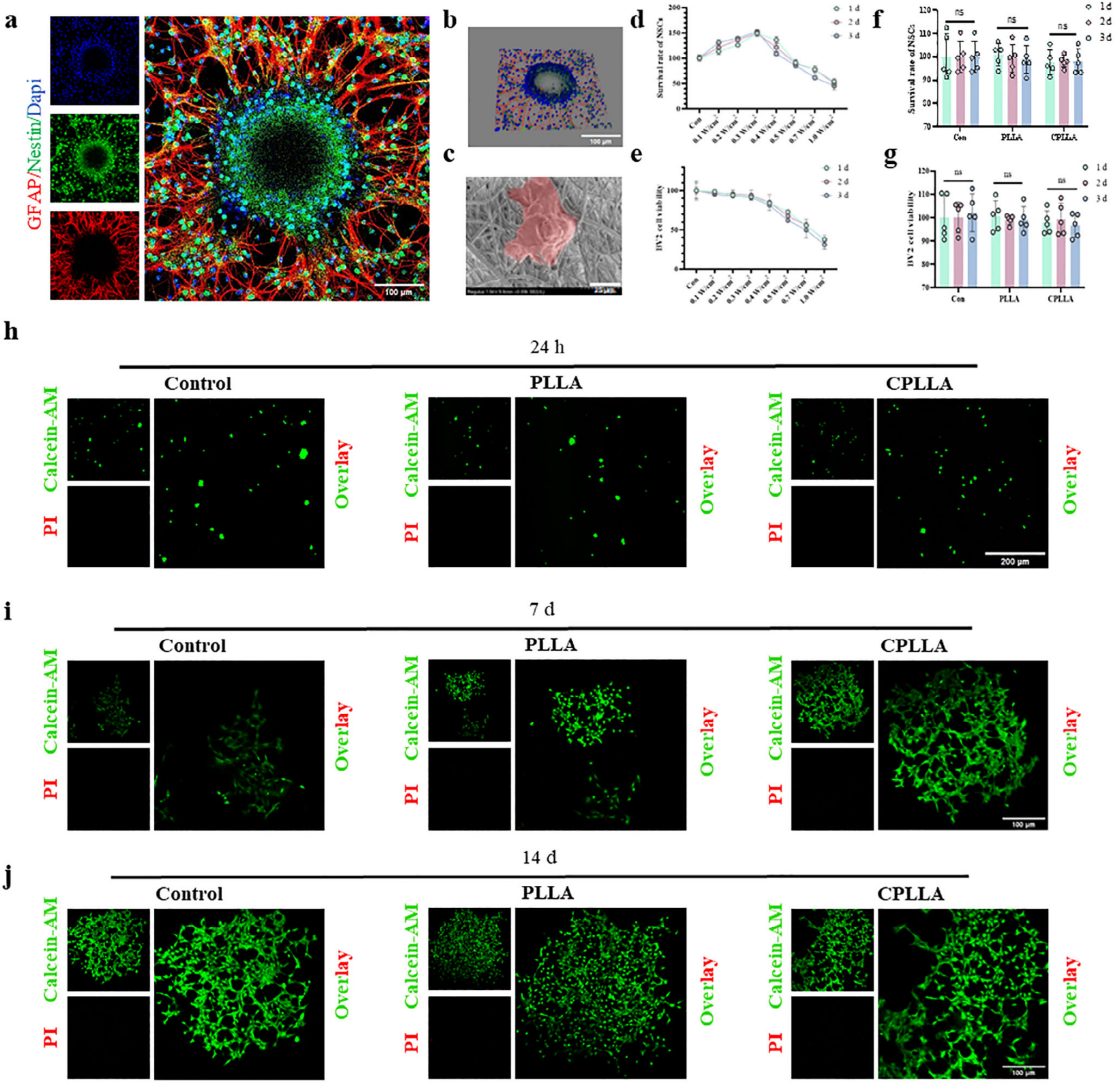
(a) Identification of NSC spheroids with differentiation capability. (b) 3D image of NSC spheroids. (c) Electron micrograph of CPLLA‐loaded NSC spheroids. (d) CCK‐8 assay results for neural stem cells treated with ultrasound alone. (e) CCK‐8 assay results for BV2 cells treated with ultrasound alone. (f) CCK‐8 assay results for NSCs treated with PLLA and CPLLA. (g) CCK‐8 assay results for BV2 cells treated with PLLA and CPLLA. Cell viability/dead cell staining images of NSCs treated with PLLA and CPLLA at different time points. (h) 24 h, (i) 7 days, (j) 14 days. Control group: 0 W/cm^2^; no material/no extract.

Prior to experimental use, NSCs were authenticated by morphological assessment and immunocytochemical verification of canonical markers, including Nestin (a cytoskeletal protein indicative of neural progenitor status) and glial fibrillary acidic protein (GFAP), which is associated with astroglial differentiation potential [33]. CLSM revealed the expected bipolar/stellate morphology and neurosphere formation under proliferative conditions, while immunostaining confirmed robust expression of Nestin and low‐level expression of GFAP, consistent with an undifferentiated NSC phenotype (Figure 3a,b). To ensure that the patch and ultrasonic stimulation parameters were compatible with both NSCs and microglial‐like BV2 cells, we not only observed the adhesion capacity of NSCs to CPLLA via scanning electron microscopy (Figure 3c), but also systematically evaluated in vitro cell compatibility. Using the Cell Counting Kit‐8 (CCK‐8) assay, we first assessed the potential cytotoxicity of PLLA and CPLLA extracts on NSCs and BV2 cells, and concurrently determined a suitable range of ultrasonic intensities for subsequent experiments. As illustrated in Figure 3d,e, when ultrasound was applied at a fixed frequency of 1.0 MHz and a duty cycle of 50%, a significant decline in cell viability was observed in both cell types once the acoustic intensity exceeded 0.3 W/cm^2^. Based on these findings, 0.3 W/cm^2^ was selected as the optimal and biocompatible ultrasonic intensity for all subsequent stimulation experiments involving PLLA and CPLLA substrates. Importantly, under these conditions, both NSCs and BV2 cells maintained normal viability when cultured in the presence of PLLA or CPLLA extracts (Figure 3f,g), indicating negligible extract‐induced cytotoxicity. To further validate these findings, live/dead cell staining experiments were conducted after culturing NSCs in PLLA or CPLLA extracts for 24 h, 7, or 14 days. Compared to the control group (0 W/cm^2^; no material/extract), staining results showed no significant increase in cell death, indicating that neither material extract adversely affected cell viability (Figure 3h–j). Concurrently, BV2 cells co‐cultured for 24 h under different treatment conditions demonstrated good cell compatibility (Figure S10).

### 2.5 PLLA and CPLLA‐Mediated Electrical Stimulation Promotes the Differentiation of Neural Stem Cells Into Neurons

**FIGURE 4:**
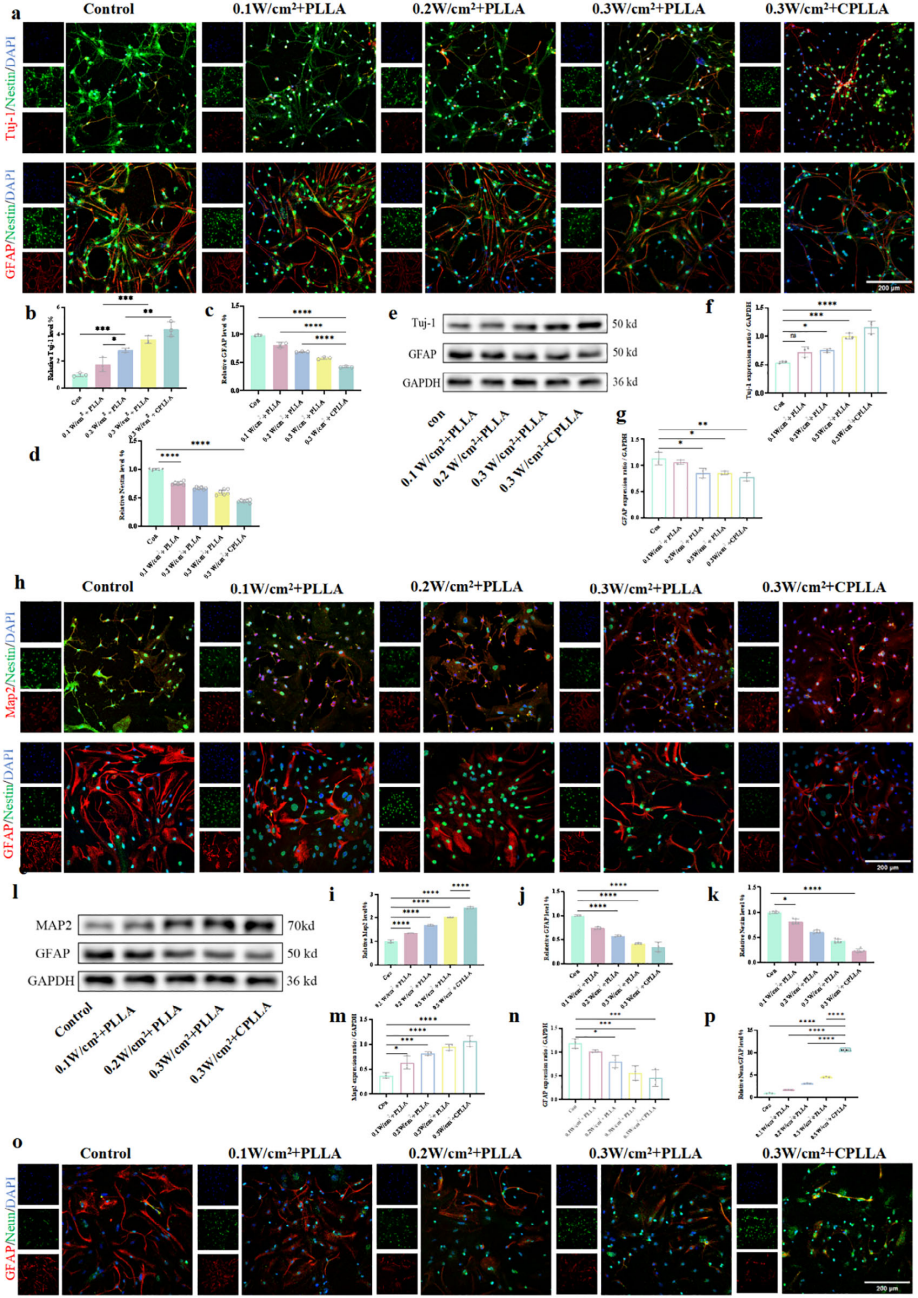
(a) Fluorescent images depicting the differentiation of NSCs following 7 days of treatment under different conditions. (b) Fluorescence intensity of Tuj‐1, (c) GFAP, and (d) Nestin in NSCs after 7 days of differentiation. (e) Western blot images of NSCs after 7 days of differentiation under the indicated treatments. (f) Western blot quantification of Tuj‐1 expression. (g) Western blot quantification of GFAP expression. (h) Fluorescent images of NSC differentiation after 14 days of treatment across different groups. Fluorescence intensity of (i) Map2, (j) GFAP, and (k) Nestin in NSCs after 14 days of differentiation. (l) Western blot images of NSCs after 14 days of differentiation under various conditions. (m) Western blot quantification of Map2 expression. (n) Western blot quantification of GFAP expression. (o) Fluorescent images showing the proportion of neuron‐like/astrocyte‐like cells after 14 days of NSC differentiation under different treatments. (p) Fluorescence intensity ratio of Neun to GFAP 14 days after neural stem cell differentiation. Data are presented as mean ± s.d., n = 3 independent biological replicates. Statistical analysis was performed using one‐way ANOVA followed by multiple comparison tests (NS, not significant; ^**^ *P* < 0.01, ^***^ *P* < 0.001, ^****^ *P* < 0.0001).

**FIGURE 5:**
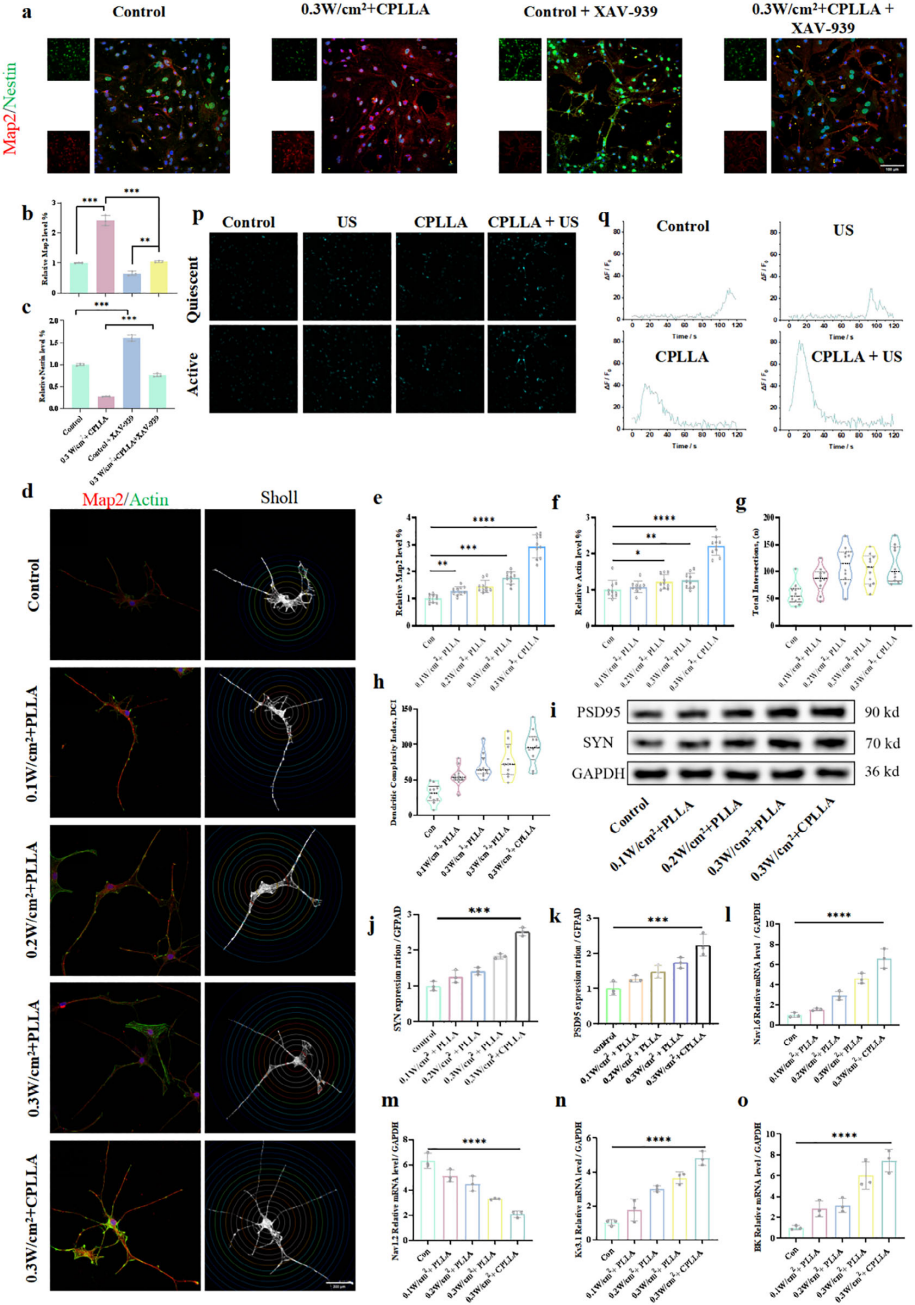
(a) Representative fluorescence images showing neuronal‑like differentiation of neural stem cells (NSCs) under XAV‑939 intervention across experimental groups at day 14. (b) Quantification of MAP2 fluorescence intensity. (c) Quantification of Nestin fluorescence intensity. (d) Sholl analysis of differentiated neurons under various conditions (n = 10). (e) MAP2 fluorescence intensity in individual neurons. (f) Actin fluorescence intensity in individual neurons. (g) Total number of intersections per neuron. (h) Neuronal complexity index. (i) Western blot analysis of NSC differentiation at day 14 (n = 3). (j) Expression level of SYN protein. (k) Expression level of PSD95 protein. Relative mRNA expression levels of (l) Nav1.6, (m) Nav1.2, (n) Kv3.1, and (o) BK (n = 3). (p) Intracellular calcium signaling imaging after 14 days of treatment under different conditions. (q) Peak calcium amplitude in each group. Data are presented as mean ± SD. Statistical significance was evaluated by one‑way ANOVA followed by multiple comparison tests (NS: not significant; ^*^ *P* < 0.05, ^**^ *P* < 0.01, ^***^ *P* < 0.001, ^****^ *P* < 0.0001).

PLLA or CPLLA were utilized as patches to deliver wireless electrical stimulation to NSCs under ultrasonication, thereby promoting their neuronal differentiation. The co‐cultured NSCs were then exposed to 2 min of cyclic ultrasonication on a daily basis. To evaluate the potential of the generated electrical signals to enhance neuronal differentiation in NSCs, immunofluorescence staining and Western Blot analysis were utilized to assess the expression of neuronal markers in the NSCs. Nestin is widely recognized as a key marker protein for neural stem cells, and thus holds paramount significance in this context [34]. β‐tubulin III (Tuj‐1), a protein that is present in neurons, serves as a marker for early‐stage neurons [35]. Microtubule‐associated protein 2 (Map2), a key protein involved in neurogenesis and microtubule assembly, is commonly used together with neuronal nuclei antigen (Neun) as important markers for mature neurons [36]. It has been employed as a marker for mature neurons. The protein known as glial fibrillary acidic protein (GFAP), which is primarily located in astrocytes, provides a reliable indicator of glial cell presence [37]. The results of the 7‐day immunofluorescence analysis indicate that neither PLLA nor CPLLA patches alone, nor exposure to 0.1,0.2, or 0.3 W/cm^2^ for 7 days, influenced NSC differentiation (Figure S11a–e). However, PLLA and CPLLA patches, when subjected to ultrasonic stimulation for 7 days, generated radiofrequency stimulation that promoted the differentiation of NSCs into neurons (Figure 4a). PLLA treated at 0.1, 0.2, and 0.3 W/cm^2^ exhibited Tuj‐1 expression levels 1.77‐fold, 2.83‐fold, and 3.60‐fold higher than the control group, respectively. Similarly, CPLLA treated at 0.3 W/cm^2^ showed Tuj‐1 expression 4.38‐fold higher than the control (Figure 4b). Concurrently, GFAP expression in PLLA treated at 0.1, 0.2, and 0.3 W/cm^2^ was downregulated by 18.89%, 31.57%, and 42.36%, respectively, compared to the control group. Furthermore, CPLLA treated at 0.3 W/cm^2^ exhibited a 58.05% reduction in GFAP expression (Figure 4c) and a 65.91% decrease in Nestin expression (Figure 4d) compared to the control group. Western blot analysis further corroborated these findings (Figure 4e–g). Fluorescence imaging after 14 days of electrical stimulation indicated (Figure 4h; Figure S11f–j) that neither PLLA nor CPLLA patches alone, nor 0.1, 0.2, or 0.3 W/cm^2^ stimulation, influenced NSC differentiation. However, 0.1, 0.2, and 0.3 W/cm^2^ exhibited Map2 expression levels 1.35‐fold, 1.68‐fold, and 2.03‐fold higher than the control group, respectively. Under 0.3 W/cm^2^ stimulation, CPLLA showed a 2.43‐fold increase in Map2 expression compared to the control (Figure 4i). Under 0.1, 0.2, and 0.3 W/cm^2^ irradiation, GFAP expression in PLLA was downregulated by 26.05%, 42.52%, and 58.50%, respectively, compared to the control group. At 0.3 W/cm^2^, CPLLA exhibited a 65.66% reduction in GFAP expression compared to the control group (Figure 4j), alongside an 86.88% decrease in Nestin expression (Figure 4k). Western blot analysis further corroborated these findings (Figure 4l–n). More significantly, after 14 days of intervention, the Neun/GFAP ratio in PLLA‐treated groups at 0.1, 0.2, and 0.3 W/cm^2^ was increased by 0.70‐fold, 2.09‐fold, and 3.57‐fold, respectively, compared to the control group. Furthermore, the Neun/GFAP ratio in CPLLA exposed to 0.3 W/cm^2^ was 9.60‐fold higher than that in the control group (Figure 4o,p), further supporting the preferential induction of neuronal lineage commitment under the combined influence of material and ultrasound stimulation. It has been demonstrated that ultrasound‐mediated piezoelectric effects promote the differentiation of neural stem cells toward the neuronal lineage through electrical stimulation. By detecting the expression levels of neuron‐specific proteins, it was found that 14 days of electrical stimulation mediated by CPLLA patches at an acoustic intensity of 0.3 W/cm^2^ increased the differentiation rate by 2.43‐fold relative to normal levels. Additionally, to validate the enhancement of neurogenesis at an earlier differentiation stage, we examined the expression level of Doublecortin (DCX), a microtubule‐associated protein specifically expressed in newborn neurons and closely associated with neuronal migration. Experimental results showed that both the number and fluorescence intensity of DCX‐positive cells were significantly increased in the ultrasound‐activated CPLLA treatment group compared to the control group (Figure S12). This finding further confirms that the intervention strategy not only promotes the recruitment and directed differentiation of endogenous neural stem cells but also effectively accelerates their conversion into early neuronal lineages, thereby establishing a cellular foundation for subsequent maturation and functional integration. Subsequently, we further explored the mechanism by which electrical stimulation promotes the differentiation of neural stem cells into neurons. RNA sequencing analysis revealed that volcano plot analysis showed 57 genes were downregulated and 71 genes were upregulated in the 0.3 W/cm^2^ + CPLLA treatment group compared to the control group (Figure S13a). GO analysis indicated significant enrichment in pathways including “neurogenesis,” “neuronal differentiation,” and “neurological development” (Figure S13b). KEGG analysis indicated significant activation of the “Wnt signaling pathway” in the differentiation group (Figure S13c). This aligns with previous studies revealing the core role of the Wnt signaling pathway in neurodevelopment and stem cell fate determination [38]. We conducted a functional validation experiment for direct verification. Four experimental groups were established: 1) Control: NSCs cultured under standard conditions; 2) 0.3 W/cm^2^ + CPLLA; 3) Inhibitor control: NSCs cultured with the Wnt‐specific inhibitor XAV‐939 in the medium; 4) 0.3 W/cm^2^ + CPLLA + XAV‐939. Neuronal differentiation was assessed by immunofluorescence staining after 14 days of culture (Figure 5a). Consistent with earlier findings, compared with the Control group, the 0.3 W/cm^2^ + CPLLA group showed a significant increase in fluorescence intensity of the mature neuronal marker Map2. The addition of XAV‐939 markedly suppressed the basal differentiation capacity of NSCs. Furthermore, compared with the 0.3 W/cm^2^ + CPLLA group, the 0.3 W/cm^2^ + CPLLA + XAV‐939 group exhibited a significant blockade of the electrical stimulation‑induced neuronal differentiation, returning to levels comparable to those of the Control or Inhibitor control groups (Figure 5b,c). These results indicate that specific inhibition of the Wnt signaling pathway effectively abolishes the pro‐neuronal differentiation effect induced by “0.3 W/cm^2^ + CPLLA.”

### 2.6 PLLA and CPLLA‐Mediated Electrical Stimulation Accelerates Neuronal Maturation

To more comprehensively evaluate the effects of electrical stimulation on neuronal complexity and maturity, we performed Sholl analysis on individual neurons (n = 10) by quantifying the formation of primary dendritic branches (Map2) and the dynamic remodeling of growth cones (F‐actin redistribution), alongside calculating the number of intersections between dendritic branches and concentric circles centred on the soma at specified radii [39]. As demonstrated by immunofluorescence images (Figure S14a–c), neither PLLA nor CPLLA patches alone, nor electrical stimulation at 0.1, 0.2, or 0.3 W/cm^2^, affected Map2 or F‐actin expression levels compared to the control group. Map2 expression in PLLA under 0.1, 0.2, and 0.3 W/cm^2^ was 1.28‐fold, 1.45‐fold, and 1.77‐fold higher than the control, respectively, while CPLLA under 0.3 W/cm^2^ showed 2.93‐fold higher expression than the control (Figure 5d,e). Regarding dynamic reconstruction of neuronal growth cones, F‐actin expression in PLLA under 0.1, 0.2, and 0.3 W/cm^2^ was 1.08‐fold, 1.22‐fold, and 1.24‐fold that of the control group, respectively. CPLLA under 0.3 W/cm^2^ exhibited 2.21‐fold higher expression than the control (Figure 5f). Sholl analysis indicated that neither the control (con) group, nor PLLA or CPLLA patch alone, nor 0.1, 0.2, or 0.3 W/cm^2^ exposure affected neuronal complexity (Figure S14d,e). PLLA treated at 0.1, 0.2, and 0.3 W/cm^2^ exhibited 1.45‐fold, 1.77‐fold, and 1.89‐fold increases in total crossings relative to the control, respectively, while CPLLA at 0.3 W/cm^2^ showed a 1.92‐fold increase in total crossings (Figure 5g). PLLA treated at 0.1, 0.2, and 0.3 W/cm^2^ exhibited complexity indices (DCI) 1.84‐fold, 2.29‐fold, and 2.55‐fold higher than the control group, respectively, while CPLLA treated at 0.3 W/cm^2^ showed a DCI 3.19‐fold higher than the control (Figure 5h).

Furthermore, we comprehensively evaluated neuronal maturation from both structural/functional and key electrophysiological perspectives. The maturation of excitatory postsynaptic densities (PSD‐95) and the density/functional status of presynaptic vesicles (SYN) collectively reflect the maturity of functional synaptic connections between neurons [40]. Western blot analysis revealed that, under 0.1, 0.2, and 0.3 W/cm^2^ conditions, the expression levels of PSD‐95 and SYN in the PLLA group increased by 1.28‐ and 1.26‐fold, 1.48‐ and 1.41‐fold, and 1.74‐ and 1.83‐fold, respectively, compared to the control. Notably, at 0.3 W/cm^2^, CPLLA induced even greater upregulation, with PSD‐95 and SYN expression reaching 2.24‐ and 2.52‐fold of the control levels (Figure 5i–k). Finally, we assessed the mRNA expression of voltage‐gated sodium channel subtypes (Nav1.2, Nav1.6), voltage‐gated potassium channels (Kv3.1), and large‐conductance calcium‐activated potassium channels (BK). The expression level and proper localization of Nav1.6 at the axon initial segment represent a gold standard for evaluating neuronal structural and functional polarization maturity, with high Nav1.6 expression and precise positioning being hallmarks of advanced maturation [41]. During embryonic and early postnatal stages, Nav1.2 is the predominant sodium channel subtype in the brain, responsible for initiating neuronal electrical signaling and is critical for action potential generation in immature neurons [42]. Thus, the dynamic shift in the Nav1.2/Nav1.6 expression ratio serves as a key molecular indicator of electrophysiological maturation [43, 44]. Kv3.1 is essential for assessing the functional maturity of specific neuronal subclasses, particularly fast‐spiking interneurons [45]. Similarly, BK channels are not only a key marker of neuronal functional maturity but also central regulators of neuronal excitability, signaling precision, and plasticity [46]. qPCR results demonstrated that, compared to the control, the 0.3 W/cm^2^ + CPLLA group induced the most pronounced upregulation of *SCN8A* (Nav1.6) gene expression (6.78‐fold increase), accompanied by a relative downregulation of *SCN2A* (Nav1.2) by 66.77%, resulting in the highest Nav1.6/Nav1.2 expression ratio among all groups (Figure 5l,m). The expression levels of *KCNC1* (Kv3.1)‐a gene associated with the fast‐spiking phenotype of mature neurons‐and *KCNMA1* (BK channels), which regulates action potential repolarization and after‐hyperpolarization, were both maximally and synergistically upregulated in the 0.3 W/cm^2^ + CPLLA group, increasing by 3.79‐ and 7.43‐fold, respectively (Figure 5n,o). These findings indicate that radiofrequency stimulation mediated by PLLA and CPLLA effectively enhances neuronal complexity and accelerates maturation processes. Finally, to assess the functional maturation of differentiated neurons, we measured dynamic changes in intracellular calcium concentrations using Fluo‐4 AM calcium indicator. On day 14 of differentiation, confocal microscopy imaging revealed that the piezoelectric group (CPLLA + US), which received combined ultrasound stimulation for 14 days, exhibited more active spontaneous calcium signaling compared to the control group, ultrasound‐only group, and CPLLA group (Figure 5p). Most cells in the control group remained quiescent or displayed only sporadic, low‐amplitude calcium fluctuations. In contrast, cells in the piezoelectric stimulation group showed prominent repetitive spontaneous calcium transients, characterized by typical rapid rise and exponential decay kinetics‐a hallmark firing pattern of mature neurons (Movies S2–S5). △F/F_0_ Signal analysis of representative cells further indicated that the mean calcium peak amplitude in the piezoelectric group was significantly higher than that in the control group (Figure 5q), suggesting that the endogenous electric field induced by the piezoelectric effect effectively promotes the establishment of cellular excitability.

### 2.7 PLLA and CPLLA‐Mediated Electrical Stimulation Alleviates LPS‐induced Neuroinflammation

**FIGURE 6:**
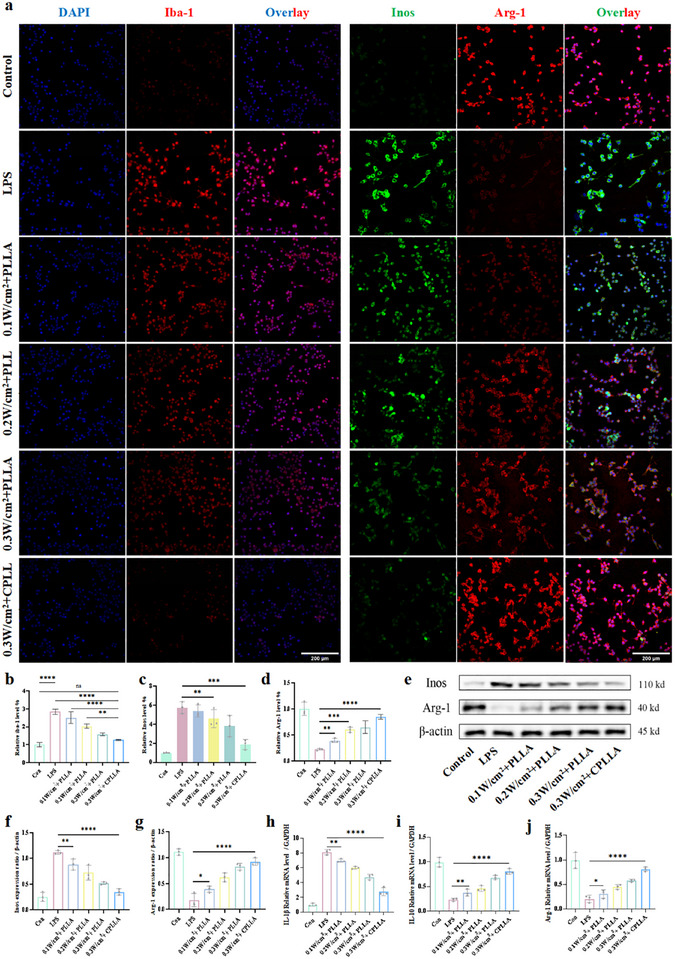
(a) Representative immunofluorescence images showing the activation and polarization states of BV2 cells under different conditions following LPS stimulation. Quantitative analysis of fluorescence intensity for (b) Iba‑1, (c) Inos, and (d) Arg‑1. (e) Western blot analysis of polarization‑related proteins in BV2 cells after 6 h of LPS stimulation under various conditions. (f) Expression levels of Inos protein. (g) Expression levels of Arg‑1 protein. Relative mRNA expression of (h) IL‑1β, (i) IL‑10, and (j) Arg‑1. Data are presented as mean ± SD (n = 3 independent biological replicates). Statistical significance was evaluated by one‑way ANOVA with multiple comparison tests (NS: not significant; ^*^ *P* < 0.05, ^**^ *P* < 0.01, ^***^ *P* < 0.001, ^****^ *P* < 0.0001).

The inflammatory immune microenvironment following TBI plays a crucial dual role in both secondary neurological damage and subsequent repair processes. During the acute phase of TBI, neurons and axons undergo progressive death, whilst vascular endothelial structures sustain varying degrees of disruption [47]. These pathological alterations facilitate the infiltration of blood components, including peripheral immune cells, into the brain parenchyma, thereby establishing a pro‐inflammatory microenvironment [48, 49]. The dynamic evolution of the immune microenvironment exerts a significant influence on the progression of acute pathologies and, moreover, determines long‐term neurological outcomes [50]. Microglia are resident macrophages of the central nervous system, playing a crucial role in immune surveillance, homeostasis, and neuroinflammation [51]. Therefore, the present study employed LPS‐induced BV2 cell lines to establish an in vitro inflammatory model. PLLA and CPLLA were used as patches to deliver wireless electrical stimulation to LPS‐induced BV2 cells for a period of 8 h under ultrasound guidance. The effects of electrical stimulation on the immune microenvironment were observed using immunofluorescence and Western blot techniques. Immunofluorescence analysis confirmed that neither the presence of PLLA or CPLLA patch alone, nor US stimulation by itself, significantly influenced BV2 cell activation (as indicated by Iba‐1 expression) or affected the polarization balance between the pro‐inflammatory M1 phenotype (marked by Inos) and the anti‐inflammatory M2 phenotype (marked by Arg‐1) (Figure S14f–i) [52]. In contrast, when LPS‐induced BV2 cells were subjected to US stimulation at varying intensities in the presence of PLLA or CPLLA patch, a clear immunomodulatory effect was observed. As shown in Figure 6a–d, both materials significantly reduced microglial activation, suppressed the expression of the M1 marker Inos, and enhanced the expression of the M2 marker Arg‐1, collectively promoting a shift from the pro‐inflammatory M1 state toward the anti‐inflammatory M2 phenotype. These immunofluorescence findings were further validated by Western blot analysis, which consistently demonstrated a downregulation of Inos and upregulation of Arg‐1 (Figure 6e–g). To further evaluate the key regulatory role of ultrasound combined with PLLA or CPLLA patches on the inflammatory response in the post‐injury neural regeneration microenvironment, we assessed the expression of phenotypic marker cytokines in microglia via qPCR (Table S2). Compared with the control group, the 0.3 W/cm^2^ + CPLLA treatment group showed significant suppression of the classical M1 phenotype cytokine IL‐1β, with its mRNA expression reduced by 65.68% (Figure 6h). Conversely, the expression of M2 phenotype markers IL‐10 and Arg‐1 was markedly upregulated in the 0.3 W/cm^2^ + CPLLA group, increasing by 2.47‐fold and 2.97‐fold, respectively (Figure 6i,j). Notably, when PLLA patches were applied, elevating the ultrasound intensity from 0.1 W/cm^2^ to 0.3 W/cm^2^ led to a graded increase in IL‐10 and Arg‐1 expression and a corresponding graded decrease in IL‐1β. More importantly, at an ultrasound intensity of 0.3 W/cm^2^, CPLLA patches elicited a significantly stronger upregulation of IL‐10 and Arg‐1 and a more pronounced downregulation of IL‐1β than did PLLA patches. This distinct gene expression profile confirms that the combined treatment regimen possesses clear anti‐inflammatory and pro‐repair immunomodulatory functions, which are conducive to establishing a supportive microenvironment for neural regeneration.

### 2.8 CPLLA‐Mediated Electrical Stimulation Promotes Neural Functional Recovery in Rats Following Traumatic Brain Injury

**FIGURE 7:**
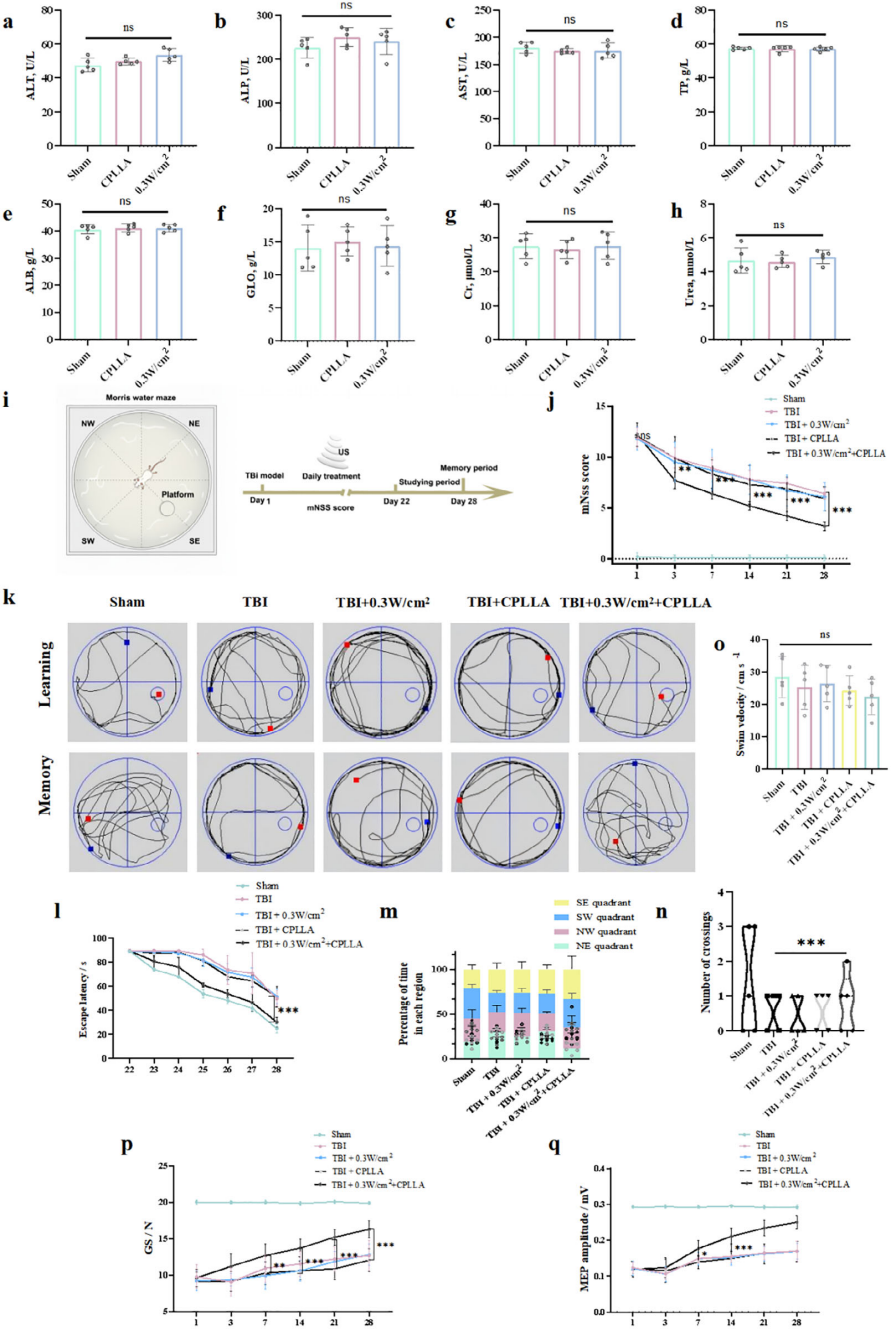
Liver and kidney function indices in rats 28 days after implantation of CPLLA or 0.3 W/cm^2^: (a) alanine aminotransferase, (b) alkaline phosphatase, (c) aspartate aminotransferase, (d) total protein, (e) albumin, (f) globulin, (g) creatinine, and (h) urea. (i) Schematic diagram of the experimental traumatic brain injury model. (j) Modified Neurological Severity Score (mNSS) recorded on days 1, 3, 7, 14, 21, and 28. (k) Representative swimming trajectories in the Morris water maze. (l) Escape latency, (m) percentage of time spent in each quadrant, (n) number of platform crossings, and (o) swimming speed. (p) Grip strength (GS) recorded on days 1, 3, 7, 14, 21, and 28. (q) Motor evoked potentials (MEP) recorded on days 1, 3, 7, 14, 21, and 28. Data were analyzed by one‐way ANOVA with multiple comparisons (NS: not significant; ^*^ *P* < 0.05, ^**^ *P* < 0.01, ^***^ *P* < 0.001).

Prior to in vivo functional assessment, we systematically evaluated the effects of ultrasound and CPLLA patches on in vivo biosafety in rats within 28 days post‐traumatic brain injury (TBI). Histopathological examination of major organs, including lungs, liver, kidneys, and spleen, revealed no morphological abnormalities or tissue damage in experimental rats implanted with CPLLA patches, indicating the absence of systemic toxicity (Figure S15). Furthermore, comprehensive serum biochemical analysis revealed no significant differences in key functional markers between the CPLLA group and the 0.3 W/cm^2^ group compared to the sham‐operated group (Figure 7a–h), collectively confirming the high biocompatibility and biosafety of the implanted material. Subsequently, we employed a multimodal strategy to evaluate the efficacy of CPLLA‐based piezoelectric intervention for neural repair. Figure 7i illustrates a schematic diagram of CPLLA patch treatment for TBI. We first investigated whether ultrasound‐driven electrical stimulation generated by CPLLA could promote motor function recovery and mitigate structural damage after TBI. Assessment using the modified Neurological Severity Score (mNSS) revealed that all TBI rats showed similarly elevated scores on day 1 post‐injury. However, from day 3 to day 28, the mNSS scores in the 0.3 W/cm^2^ + CPLLA group were significantly lower than those in the untreated TBI group, the ultrasound‐alone (0.3 W/cm^2^) group, and the CPLLA‐alone group, indicating sustained functional recovery (Figure 7j). To further evaluate cognitive restoration, the Morris water maze test was conducted (Figure 7k). During the search phase after TBI, rats in the 0.3 W/cm^2^ + CPLLA group exhibited a significantly shorter escape path length compared to the TBI group, the ultrasound‐alone group, and the CPLLA‐alone group, suggesting enhanced spatial learning ability (Figure 7l). Moreover, in the memory phase, the 0.3 W/cm^2^ + CPLLA group demonstrated significantly superior performance across multiple cognitive metrics compared to the TBI, ultrasound‐alone, and CPLLA‐alone groups: prolonged dwell time in the target quadrant and increased number of platform crossings (Figure 7m,n), with no notable difference in swimming speed observed among groups (Figure 7o). In addition, we employed electrophysiological and functional methods, including Grip Strength (GS) testing and Motor Evoked Potential (MEP) recordings, to systematically evaluate the effects of the combined 0.3 W/cm^2^ + CPLLA intervention on limb muscle strength recovery and the structural and functional integrity of motor pathways in rats following traumatic brain injury (TBI). The results indicated that, beginning from day 7 post‐injury, grip strength in the 0.3 W/cm^2^ + CPLLA group was significantly greater than that in the TBI control group, the ultrasound‐alone group, and the CPLLA‐alone group, and this advantage persisted through day 28, suggesting effective restoration of limb muscle function (Figure 7p). MEP recordings further provided electrophysiological evidence that the amplitude of motor evoked potentials was significantly increased in the 0.3 W/cm^2^ + CPLLA group from day 7 onward, indicating improved neural excitability in the central motor conduction pathway (Figure 7q). Together, these findings demonstrate that the combined 0.3 W/cm^2^ + CPLLA intervention synergistically promotes both peripheral muscle recovery and the functional restoration of central motor pathways. These behavioral and electrophysiological improvements collectively highlight the positive role of 0.3 W/cm^2^ ultrasound combined with CPLLA‐mediated electrical stimulation in facilitating motor and cognitive rehabilitation after neural injury.

### 2.9 CPLLA‐Mediated Electrical Stimulation Promotes Tissue Remodeling in Rats Following Traumatic Brain Injury

**FIGURE 8:**
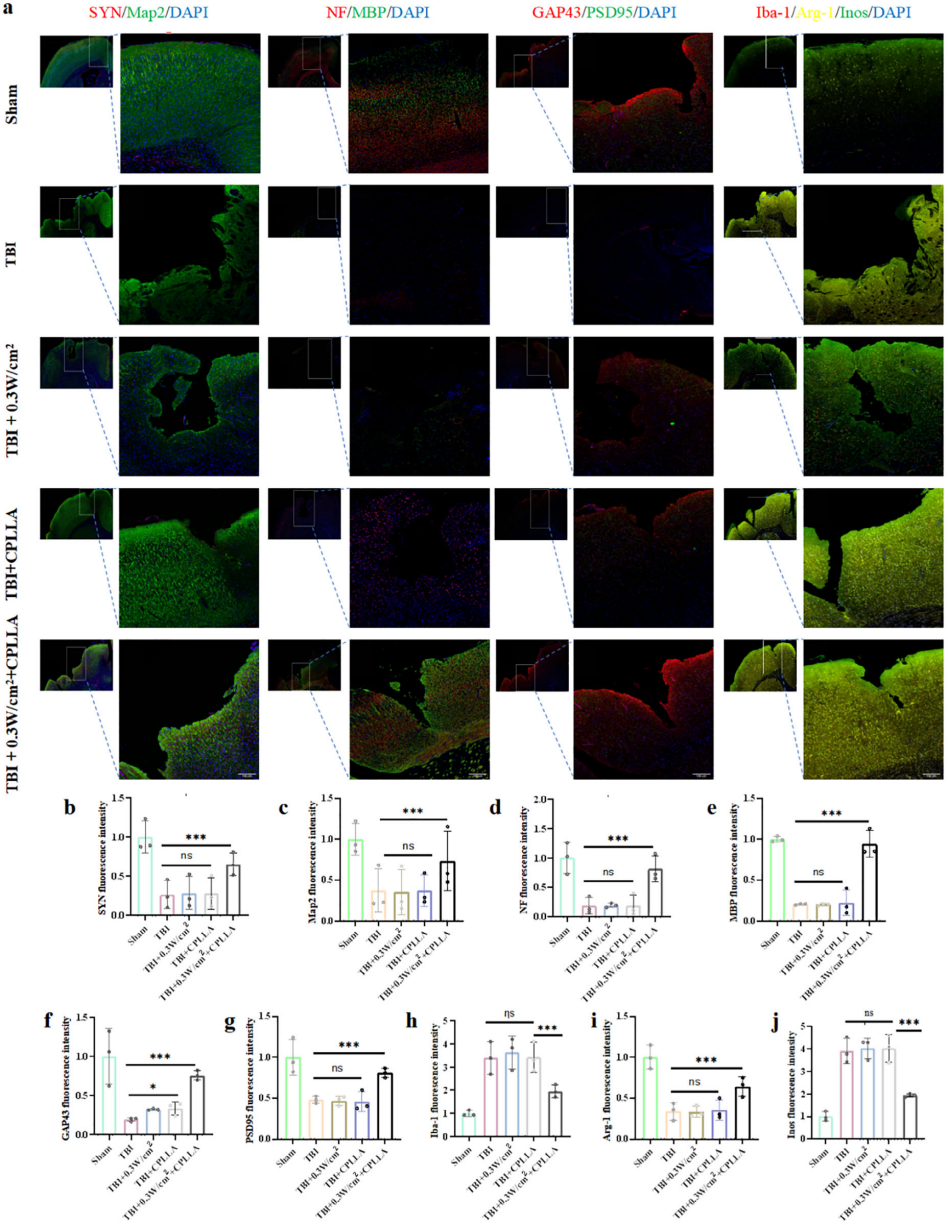
(a) Representative immunofluorescence images of brain sections from rats 28 days post‐TBI showing staining for SYN/Map2, NF/MBP, GAP43/PSD95, Iba‐1, Arg‐1, and iNOS. Quantitative analysis of each marker (n = 3).(b) SYN expression. (c) Map2 expression. (d) NF expression. (e) MBP expression. (f) GAP43 expression. (g) PSD95 expression. (h) Iba‐1 expression. (i) Arg‐1 expression. (j) iNOS expression. Data were analyzed by one‐way ANOVA followed by multiple comparisons (NS: not significant; ^*^ *P* < 0.05, ^**^ *P* < 0.01, ^***^ *P* < 0.001).

To comprehensively evaluate the structural and functional recovery of neural tissue following TBI, we performed a multi‐modal histological and imaging analysis. The assessment of nerve fibre regeneration and myelination at the lesion site was conducted using dual immunofluorescence labeling for neurofilament (NF, marking axons) and myelin basic protein (MBP). Synaptogenesis and neuronal integrity were evaluated through synaptophysin (SYN) and microtubule‐associated protein 2 (Map2) double staining. Furthermore, to specifically assess neuronal network rewiring and synaptic plasticity, we employed double labeling for growth‐associated protein 43 (Gap43, indicative of axonal growth cones and regeneration) and postsynaptic density protein 95 (PSD95, a marker of mature excitatory synapses). Concurrently, to precisely delineate the functional polarization of microglia/macrophages within the inflammatory milieu, we utilized triple immunofluorescence staining against Iba‐1, inducible nitric oxide synthase (Inos, M1 pro‐inflammatory phenotype), and arginase‐1 (Arg‐1, M2 anti‐inflammatory phenotype) (Figure 8a). The integrated results demonstrated that, compared to the TBI group, the ultrasound‐alone (0.3 W/cm^2^) group, and the CPLLA‐alone group, the 0.3 W/cm^2^ + CPLLA intervention group exhibited a significant promotion of post‐TBI nerve fibre regeneration and remyelination, a marked enhancement in synaptic density and neuronal network plasticity, a substantial reduction in overall microglial activation (Iba‐1+ cells), and a distinct shift in the polarization balance from the pro‐inflammatory M1 (Inos+) state toward the reparative M2 (Arg‐1+) phenotype (Figure 8b–j).

**FIGURE 9:**
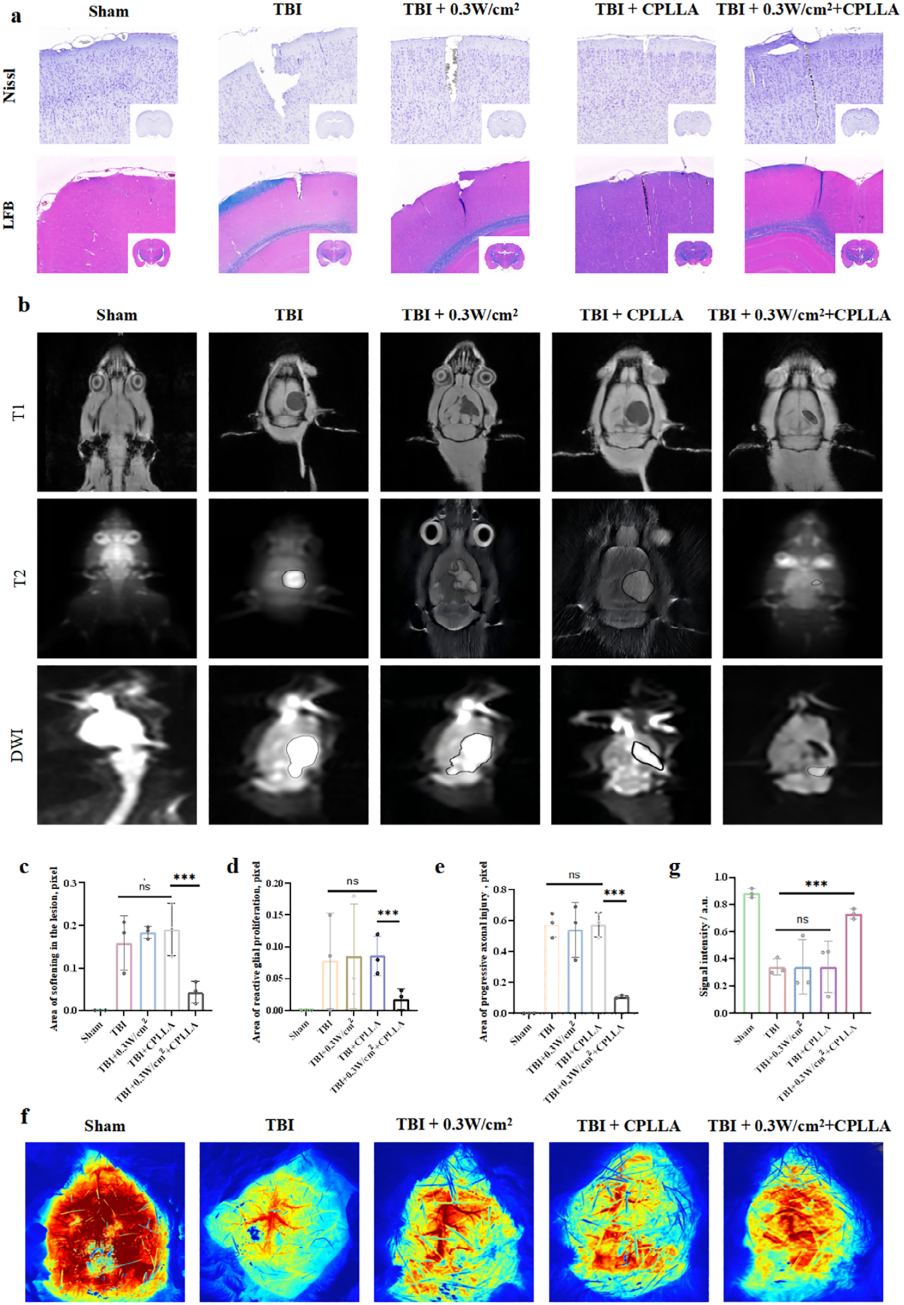
(a) Representative magnified scanned images of hematoxylin and eosin (H&E) and Nissl staining in brain tissue sections; images are shown from one rat per experimental group (n = 3). (b) Magnetic resonance imaging (MRI) of rats at 28 days post‐injury, with representative axial cross‐sectional images presented from one animal per group (n = 3). (c) Quantified lesion area, (d) reactive gliosis area, and (e) progressive axonal injury area were assessed 28 days after injury. (f) Laser speckle contrast images of brain tissue obtained from rats at 28 days post‐injury, depicting one representative animal per group (n = 3). (g) Quantitative analysis of laser speckle signal intensity. Statistical significance was evaluated using one‐way ANOVA followed by multiple comparison tests (NS, not significant; ^*^ *P* < 0.05, ^**^ *P* < 0.01, ^***^ *P* < 0.001).

Complementing these findings, Luxol Fast Blue (LFB) staining confirmed significant myelin recovery in the 0.3 W/cm^2^ + CPLLA group, in contrast to the extensive myelin loss observed in the TBI controls, the ultrasound‐alone (0.3 W/cm^2^) group, and the CPLLA‐alone group (Figure 9a). Likewise, Nissl staining revealed a higher density and more extensive distribution of Nissl bodies, indicative of robust protein synthesis and metabolic activity, in the 0.3 W/cm^2^ + CPLLA group, suggesting a significant promotion of endogenous neuronal survival and regenerative repair (Figure 9a). Further in vivo validation was obtained through magnetic resonance imaging (MRI) at 28 days post‐TBI (Figure 9b). T1‐weighted imaging visualized areas of neuronal necrosis and liquefaction, T2‐weighted imaging highlighted regions of reactive gliosis and edema, and diffusion‐weighted imaging (DWI) delineated zones of progressive axonal injury [53]. Quantitative analysis of MRI data indicated that the 0.3 W/cm^2^ + CPLLA group exhibited a significant reduction in lesion volume, attenuated cerebral edema, and enhanced tissue integrity compared to the TBI group, 0.3 W/cm^2^ group, and the CPLLA‐alone group. (Figure 9c–e). Post‐TBI astrocyte swelling has been shown to induce tissue edema, elevated intracranial pressure, and reduced cerebral blood flow perfusion. This, in turn, has been demonstrated to exacerbate ischemia and perpetuate a vicious cycle [54]. Therefore, laser speckle contrast imaging (LSCI) was employed to evaluate cerebral blood flow (CBF) dynamics (Figure 9g). The results demonstrated that the 0.3 W/cm^2^ + CPLLA group exhibited significantly improved perfusion in the perilesional area compared to the hypoperfused TBI controls, the 0.3 W/cm^2^ group, and the CPLLA‐alone group, suggesting that the piezoelectric stimulation therapy also facilitates the restoration of local microcirculation, which is critical for tissue repair (Figure 9f). In summary, the convergent evidence from histology, molecular imaging, and hemodynamic assessment robustly demonstrates that ultrasound‐driven electrical stimulation delivered by the CPLLA piezoelectric patch is a highly efficacious therapeutic strategy for promoting comprehensive neurovascular repair and functional recovery after TBI. To validate the crucial role of endogenous NSCs in promoting mature neurons, we adopted a classic lineage‐tracing strategy that combines BrdU labeling with the mature neuron‐specific marker NeuN to identify actively proliferating neuronal cells. Our analysis focused not only on the injury core region (cortex) but also specifically on the subventricular zone (SVZ), the primary niche of neural stem cells (Figure S16a). The results revealed that, compared with the TBI group, the combined ultrasound+CPLLA treatment group showed a significant increase in the number of NeuN/BrdU+ cells both in the SVZ and in the peri‐lesional cortex. In contrast, neither ultrasound alone nor CPLLA alone exhibited such an increasing trend (Figure S16b–e). Thus, lineage tracing data directly demonstrate that the combined ultrasound and CPLLA treatment markedly enhances the differentiation and maturation of endogenous neural stem cells toward the neuronal lineage (NeuN/BrdU+). We further performed Nestin/DAPI immunofluorescence staining on brain tissue sections from brain‐injured rats implanted with CPLLA for 28 days. As shown in (Figure S17), a large number of Nestin‐positive cells were observed in the tissue surrounding and within the CPLLA implant. The presence of these Nestin‐expressing cells indicates that endogenous neural stem cells are actively recruited and retained in the vicinity of the biomaterial over an extended period. These findings suggest that CPLLA not only provides a suitable microenvironment for endogenous neural stem cells but also promotes their localization to the injury site, thereby supporting its role in facilitating neurogenesis and tissue repair. This strategy of guiding the differentiation of endogenous stem cells toward neurons not only holds therapeutic potential for traumatic brain injury but may also be extended to broader applications. The electrical stimulation technology utilizing a wireless patch likewise demonstrates promise in the study of regenerative therapies for stroke, spinal cord injury, and other neurodegenerative diseases. It is important to note that, although early studies have shown that ultrasound‐guided electrical stimulation from materials can promote the differentiation of implanted NSCs into neurons, such stimulation typically relies on patches or particles fabricated from high‐performance inorganic materials [17, 55, 56, 57, 58, 59]. Moreover, these inorganic materials degrade slowly or are nearly non‑degradable in vivo, and their long‑term retention has been shown to induce chronic inflammatory responses or fibrotic encapsulation. Furthermore, transplanted NSCs face severe challenges in the lesion area: ischemia‑hypoxia, attack by inflammatory factors, and the lack of a supportive matrix, leading to extremely low post‑transplantation survival rates [60, 61]. In contrast, our strategy offers significant safety advantages over cell transplantation, as it better aligns with the regulatory mechanisms of the host brain microenvironment. By fully harnessing the inherent integration capacity of endogenous NSCs, which are perfectly compatible with the host system, our approach enables more precise and stable reconstruction of neural circuits.

### 2.10 Electrical Stimulation Promotes Neuronal Differentiation of Endogenous Neural Stem Cells via the Wnt Signaling Pathway

**FIGURE 10:**
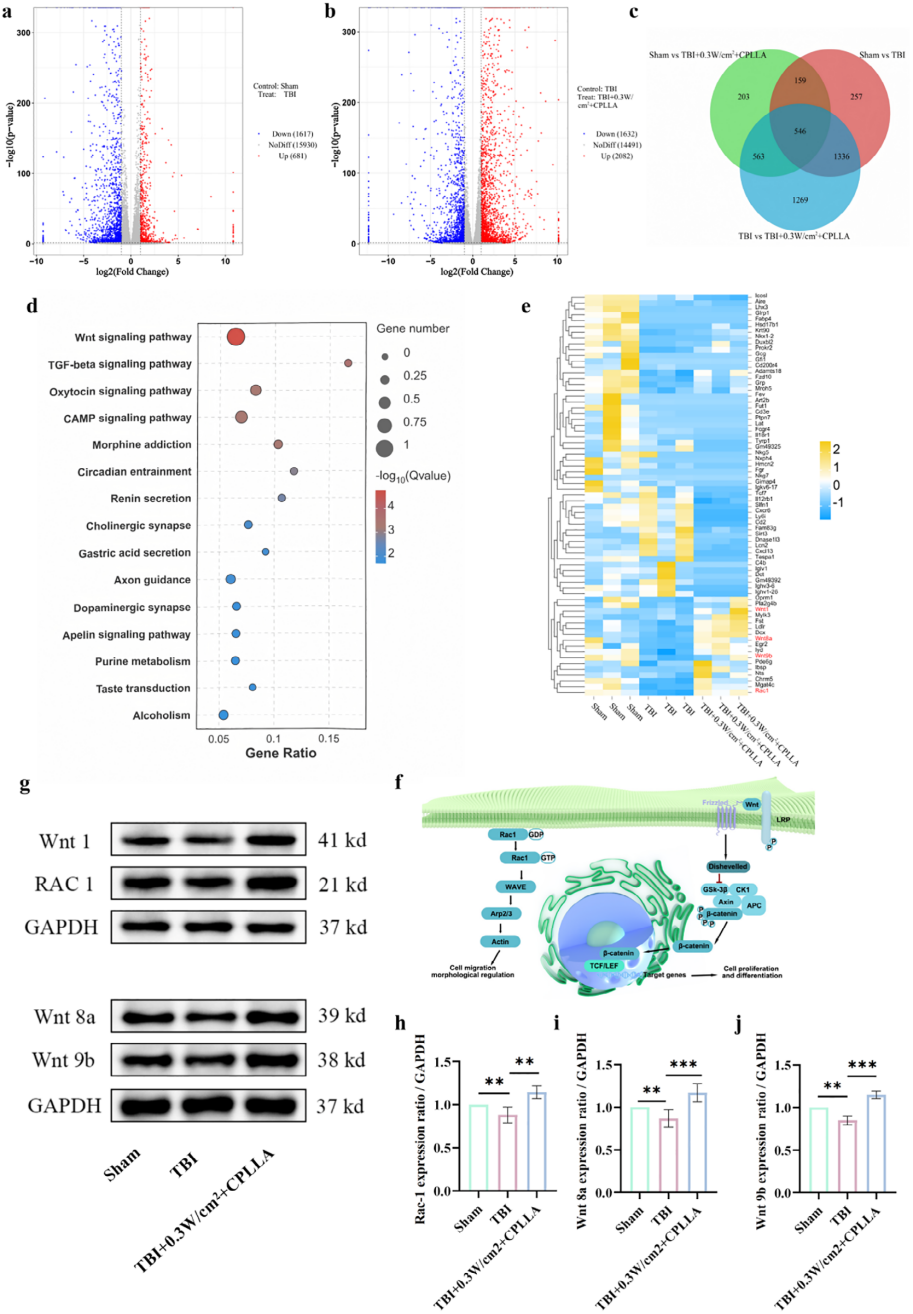
Volcano plot of differentially expressed genes (a, Sham group versus TBI group; (b) TBI group versus TBI+0.3 W/cm^2^+CPLLA group, n = 3). Red dots: Upregulated genes; green dots: Downregulated genes. (c) Number of differentially expressed genes among the three groups. (d) KEGG pathway enrichment analysis of differentially expressed genes among the three groups. (e) Gene expression heatmap of the three groups. (f) Schematic diagram of gene regulation in cell differentiation. (g) Western blot images of proteins encoded by relevant genes. h, Expression levels of (h) Rac‐1, (i) Wnt8a, and (j) Wnt9b proteins. n = 3 independent biological replicates. Data are presented as mean ± s.d. Statistical analysis performed using one‐way ANOVA and multiple comparison tests (NS, not significant; ^*^ *P* < 0.05, ^**^ *P* < 0.01, ^***^ *P* < 0.001, ^****^ *P* < 0.0001).

In order to investigate the mechanism by which CPLLA‐mediated electrical stimulation promotes the differentiation of endogenous NSCs into neurons following TBI, we conducted an analysis of RNA sequencing. Principal component analysis revealed distinct profiles of RNA between the sham group and the TBI group, as well as between the TBI group and the TBI + 0.3 W/cm^2^ + CPLLA group, indicating significant differences following electrical stimulation therapy. Volcano plot analysis identified 2298 differentially expressed RNAs post‐TBI, comprising 681 significantly upregulated and 1617 significantly downregulated RNAs. Following electrical stimulation therapy, 3714 differentially expressed RNAs were identified, comprising 2082 significantly upregulated and 1632 significantly downregulated RNAs (Figure 10a,b). In order to provide a clear illustration of the differences in the composition of RNAs between TBI group and the TBI+0.3 W/cm^2^ + CPLLA group, a Venn diagram analysis was conducted. It is noteworthy that 546 RNAs were identified as being present in three groups (Figure 8c). Kyoto Encyclopedia of Genes and Genomes (KEGG) pathway analysis further indicated significant enrichment in pathways such as Wnt, cAMP, and Oxytocin (Figure 10d). Hierarchical clustering heatmaps revealed that genes associated with neural differentiation, including Wnt1, Wnt8a, Wnt9b, and Rac1, were significantly upregulated in the electricity‐treated group compared to the TBI group (Figure 10e). The findings suggest that 0.3 W/cm^2^ combined with CPLLA may promote neuronal differentiation and maturation of NSCs via the Wnt signaling pathway (Figure 10f). In accordance with this observation, Western blot analysis revealed significantly elevated expression of Wnt1, Wnt8a, Wnt9b, and Rac1 in electricity‐treated cells, thereby providing protein‐level validation of the results obtained from the RNA sequencing analysis (Figure 10g–j).

## 3 Conclusion

In summary, this study successfully developed a high‐performance organic wireless patch capable of delivering electrical stimulation to NSCs under external ultrasound control, significantly promoting their differentiation into functional neurons. In vivo experiments demonstrated that varying acoustic intensities effectively promoted the repair of damaged brain tissue. This wireless patch technology offers novel insights for stem cell‐based therapeutic strategies, exhibiting promising application potential in the repair of neurological injuries and neurodegenerative diseases. It is important to note that in this study, at an ultrasound intensity of 0.3 W/cm^2^, cell death occurred when the ultrasound parameters were set to a frequency of 1.0 MHz and a duty cycle of 50%. In subsequent research, the potential exists for the enhancement of stimulation efficiency and clinical applicability through the modification of ultrasound frequency, duty cycle, and intensity, in conjunction with multimodal parameters and the wireless patch.

## 4 Methods

### 4.1 Preparation and Characterization of PLLA and CPLLA

Two electrospinning protocols were employed in the synthesis of PLLA fibre membranes. The first method employed involved the dissolution of 2 g of 110 kDA PLLA (Natureworks, USA) in 20 mL of dichloromethane. The mixed solution was then injected into a plastic syringe (20 mL), which was fitted with a stainless steel needle with a passivated inner diameter of 0.58 mm. The syringe was subsequently connected to a metering pump, thereby enabling the delivery of the electrospinning solution to the needle tip at a constant rate of 2 millilitres per hour. The distance between the spinneret and the grounded cylindrical collector (wrapped in 5 cm‐wide aluminum foil) was set at 14.5 cm. Electrospinning was conducted under an electric field of 1.5 kV cm^−1^ using a high‐voltage power supply (DC voltage 20 kV, negative collector voltage −5 kV, positive needle tip voltage 16.75 kV). The collector speed was adjusted to 300 and 1600 rpm in order to produce random and oriented fibres, respectively [43]. Subsequent to a duration of 3 h of electrospinning at ambient temperature, the fibres were subjected to vacuum‐drying at 80°C for a period of 24 h, with the objective of volatilizing residual organic solvents. The second method involved altering the solvent for electrospinning, with the subsequent steps being analogous. A quantity of 110 kDA PLLA, weighing 1.6 g, was dissolved in 8 mL of hexafluoroisopropanol and 2 mL of formic acid. The mixed solution was then injected into a plastic syringe (10 mL), which was fitted with a passivated stainless‐steel needle (with an inner diameter of 0.58 mm). The assembly was mounted on a metering pump, the function of which was to deliver the electrospinning solution to the needle tip at a constant rate of 1.0 mL h^−1^. The take‐off distance between the spinneret and the grounded cylindrical collector (wrapped in 5 cm‐wide conductive aluminum foil) was set at 14.5 cm. The electrospinning high‐voltage power supply was set to 18 kV and −2 kV, with the take‐off roller speed ranging from 300 to 1600 rpm. The synthesized PLLA patch sample was placed on the metal substrate and held in place by a fixture in drying oven to 105°C and then maintained at this temperature for 600 min. After cooling to room temperature, the CPLLA film was obtained.

### 4.2 Surface Modification

In order to enhance cell adhesion to the fibre membrane, a plasma treatment strategy was employed to introduce oxygen‐containing polar groups onto the fibre surface and increase surface roughness. This process has been shown to improve the hydrophilicity and biocompatibility of the PLLA fibre membrane. Samples of thermally calcined PLLA fibre membrane were prepared by cutting them to an appropriate size (2 cm × 2 cm). These samples were then subjected to a gentle wash with anhydrous ethanol, the purpose of this being to remove surface contaminants. The samples were then left to air‐dry at room temperature. The samples were then meticulously positioned on the designated sample stage within the reaction chamber of the plasma cleaner, ensuring that the treatment surface faced upward. The chamber was sealed and evacuated to a base pressure of below 10 Pa. Subsequently, air was introduced into the chamber, with the gas flow adjusted to stabilize the working pressure between 30–50 Pa. The electrical power was set to 100 W for a treatment duration of 1 min. The plasma generator was subsequently activated in order to perform surface treatment on the samples.

### 4.3 Microstructural and Structural Characterization

SEM observation: Fibre membrane samples were mounted on conductive adhesive and subjected to gold sputtering (current 15 mA, duration 60 s). The morphology of the fibre, its diameter distribution and orientation structure were observed at an acceleration voltage of 5 kV (S‐3400N, Hitachi, Japan). The analysis was conducted using XRD (D8 Advance, Bruker, Germany). A Cu Kα radiation source (λ = 0.154 nm) was utilized to perform scans at a rate of 10° min^−1^ within the 2θ range of 5° to 90°. PFM Testing: The employment of a conductive probe is imperative for the execution of piezoelectric force spectroscopy on individual fibres in contact mode (MFP‐3D Infinity, Asylum Research, USA). An alternating bias voltage of 50 V at 300 kHz was applied, with piezoelectric amplitude and phase signals recorded to characterize the local piezoelectric constant. Macro piezoelectric output testing: A 2 cm × 2 cm fibre membrane sample was positioned between two aluminum electrodes. The electrodes were encapsulated using polyimide tape (DuPont). Exposed aluminum foil electrodes were then reinforced with copper tape. These were connected to an oscilloscope. The open‐circuit voltage and short‐circuit current, generated under varying applied forces, were recorded in real time.

### 4.4 Cell Experiments

#### 4.4.1 Isolation and Culture of Neural Stem Cells

Primary NSCs were isolated from the cortical tissue of female rat embryos at gestation day 14. The collected cells were then suspended in DMEM‐F12 complete medium (supplemented with 20 ng mL^−1^ epidermal growth factor, 20 ng mL^−1^ basic fibroblast growth factor, 2% B27 plus, 2% N_2_, 1% penicillin‐streptomycin, 2 mM glutamine, and 2 mg mL^−1^ sodium heparin). Culturing was then performed in flasks at 37°C in a 5% CO_2_ incubator. The medium was replaced in part on a three‐day cycle, with cell passaging undertaken on a seven‐day cycle. The dissociation of individual neural spheres from cell aggregates was achieved through the utilization of Accutase enzyme (Gibco), thereby facilitating the generation of new neural spheres. The identification of NSCs was conducted through the utilization of confocal microscopy (TCS SP8, Leica, Germany), and their differentiation potential was subsequently evaluated. Third‐passaged mNSCs were then employed for the subsequent cellular experiments.

#### 4.4.2 Safety of PLLA, CPLLA and Ultrasound Intensity for Neural Stem Cells

To evaluate the cytotoxicity of PLLA, CPLLA, and different ultrasound intensities in vitro, neural stem cells (NSCs) and BV2 cells were seeded in 96‐well plates at initial densities of 1 × 10^4^, 5 × 10^3^, and 1 × 10^3^ cells per well, respectively. After incubation for 24 h, 7 days, and 14 days, cells in each material group were washed with phosphate‐buffered saline (PBS). Subsequently, PLLA and CPLLA extracts were added to the appropriate wells. The ultrasonic groups were subjected to acoustic intensities of 0.1, 0.2, 0.3, 0.4, and 0.5 W/cm^2^, respectively, prior to their return to the incubator (ultrasonic stimulation parameters: The frequency was set at 1.0 MHz and the duty cycle at 50%. The process was carried out on a daily basis for a period of 2 min. The egg was then subjected to an incubation process at a temperature of 37°C for a period of 24 h. The assessment of cell viability was conducted by means of the Cell Counting Kit 8 (CCK‐8, Solabio, China), with the analysis of cell proliferation being undertaken in strict accordance with the manufacturer's protocol. Furthermore, the Calcein/PI Cell Viability and Cytotoxicity Detection Kit (Beyotime, China) was utilized to differentiate between live and dead cells. Observations were conducted under confocal fluorescence microscopy (Leica, Germany), wherein live cells exhibited green fluorescence and dead cells displayed red fluorescence.

#### 4.4.3 The Effect of Electrical Stimulation on the Differentiation of Neural Stem Cells

Immunofluorescence: A total of 3 × 10^5^ mNSCs were seeded onto 20 mm diameter confocal culture dishes, which had been pre‐coated with 0.01% poly‐D‐lysine. A series of experiments were conducted employing multiple treatment regimens, including the control group, ultrasound intensities of 0.1, 0.2, and 0.3 W/cm^2^, PLLA alone, CPLLA alone, combinations of ultrasound with PLLA (0.1 W/cm^2^ + PLLA, 0.2 W/cm^2^ + PLLA, 0.3 W/cm^2^ + PLLA), the combination of 0.3 W/cm^2^ + CPLLA, and XAV‐939 at a concentration of 1 µM. The samples were then cultured for a period of 7 and 14 days, respectively. Following fixation with 4% paraformaldehyde, the cells underwent permeabilization with 0.1% Triton X‐100 and blocking with a 10% BSA solution. Subsequently, cells were incubated overnight at 4°C with the following primary antibodies: rabbit anti‐Nestin antibody (19483‐1‐AP, Proteintech, 1:400), rabbit anti‐Neun antibody (12943, Cell Signaling Technology, 1:400), mouse anti‐GFAP antibody (3670, Cell Signaling Technology, 1:400), mouse anti‐Tuj‐1 antibody (ab78078, Abcam, 1:400), rabbit anti‐Map2 antibody (ab183830, Abcam, 1:400), followed by incubation with goat anti‐rabbit IgG H&L (Alexa Fluor 488, ab150077, Abcam, 1:200) and goat anti‐mouse IgG H&L (Alexa Fluor 594, ab150116; Abcam, 1:200) at room temperature in the dark for 1 h. Finally, cell nuclei were stained with DAPI dye, and cell morphology was observed via laser confocal microscopy.

Western blot analysis: Proteins extracted from cells within distinct groups were lysed in a solution comprising RIPA lysis buffer, protease inhibitors, and phosphatase inhibitors (Solarbio). Following lysis, proteins were collected by centrifugation and denatured using 5 × sodium dodecyl sulphate (SDS) loading buffer. Thereafter, they were separated by 10% SDS‐PAGE (Solarbio). Subsequently, proteins were transferred onto a 0.22 µm PVDF membrane (Invitrogen) and blocked with 5% BSA (Beyotime). Incubate the membrane with the primary antibody at 4°C overnight, followed by three washes with PBS. Then, incubate with the secondary antibody for one hour at room temperature. Finally, perform three more washes with PBS and expose the membrane for imaging using the ChemiDoc MP imaging system (Bio‐Rad) with chemiluminescent detection (Invitrogen).

#### 4.4.4 Analysis of Neuronal Maturity via Electrical Stimulation

Five million NSCs were seeded into 20 mm diameter confocal culture dishes and cultured for 14 days following the aforementioned interventions. Following fixation with 4% paraformaldehyde, the cells underwent permeabilization with 0.1% Triton X‐100 and blocking with 10% bovine serum albumin (BSA) solution. Subsequently, cells were incubated overnight at 4°C with mouse anti‐Map2 antibody (67015‐1‐Ig, Proteintech, 1:400) and goat anti‐mouse IgG H&L (Alexa Fluor 594, ab150116; Abcam, 1:200) and Actin‐Tracker Green‐488 (Beyotime) at room temperature in the dark for one hour. Finally, cell nuclei were stained with DAPI dye, and cell morphology was observed via laser confocal microscopy. The dendritic complexity index (DCI) and total number of synapses were analyzed using Sholl analysis (open source software, ImageJ). The Western blot method used primary antibodies: rabbit anti‐PSD95 antibody (ab18258, Abcam, 1:1000) and rabbit anti‐SYN antibody (ab64581, Abcam, 1:1000). Primers used in qPCR for SCN2A (Nav1.2), SCN8A (Nav1.6), KCNC1 (Kv3.1), and KCNMA1 (BK) (Table S1).

#### 4.4.5 Electrical Stimulation Ameliorates Neuroinflammation

A total of 3 × 10^5^ BV2 cells were seeded onto confocal culture dishes and treated with lipopolysaccharide (LPS, 1 µg ml^−1^) to simulate a TBI injury model. Following the aforementioned procedures, cells from each group were fixed with 4% paraformaldehyde after 8 h, permeabilized with 0.1% Triton X‐100, and blocked with 10% BSA solution. Subsequently, the cells were incubated overnight at 4°C with the following primary antibodies: mouse anti‐Iba‐1 antibody (ab283319, Abcam, 1:400), rabbit anti‐Inos antibody (18985‐1‐AP, Proteintech, 1:400) and mouse anti‐Arg‐1 antibody (66129‐1‐Ig, Proteintech, 1:400). These were then incubated with goat anti‐rabbit IgG H&L (Alexa Fluor 488, ab150077, Abcam, 1:200) and goat anti‐rabbit IgG H&L (Alexa Fluor 594, ab150116; Abcam, 1:200) at room temperature for one hour. Finally, cell nuclei were stained with DAPI dye, and cell morphology was observed via laser confocal microscopy. Protein expression levels of Inos and Arg‐1 were again analyzed by Western blot. Primers used in qPCR for IL‐10, Arg‐1, and IL‐1β (Table S1).

### 4.5 Animal Experiments

#### 4.5.1 Establishment of TBI Models and Membrane Implantation

A standardized impact method was employed to establish the traumatic brain injury (TBI) model. Briefly, male rats under anesthesia were immobilized in a stereotaxic apparatus (68507, RWD, China), and brain injury was induced by precisely controlling the impact force and angle. Subsequently, a pre‐prepared patch material was implanted into the lesion area. Postoperative care and interventions were routinely administered, and behavioral and physiological parameters of the rats were regularly monitored to evaluate the effect of the patch on neural repair. Specifically, the rats were randomly assigned to three groups: Sham, TBI, 0.3 W/cm^2^, CPLLA and 0.3 W/cm^2^ + CPLLA (n = 40 per group). The rats were identified using ear tags, and the study was conducted in a blinded manner for both group allocation and quantitative analysis. The procedure began with a 4‐h fasting period (with free access to water), followed by anesthesia induction via inhalation of isoflurane (3.5% for induction, 2.0% for maintenance) to ensure adequate anesthesia throughout the procedure. Respiration, heart rate, and body temperature were monitored during anesthesia maintenance. A circular cranial window 10 mm in diameter was then created using a drill over the left parietal region of the skull (2.2 mm posterior to the coronal suture and 3.5 mm lateral to the sagittal suture). A controlled cortical impact device was used to induce injury (parameters: depth 4 mm, impact velocity 5 m s^−1^, dwell time 12 ms). Hemostasis during surgery was achieved by topical application of thrombin (50‐1000 U mL^−1^) (Solarbio, Beijing, China). The group receiving implantation of sterile CPLLA into the injured cortex was designated as the 0.3 W/cm^2^ + CPLLA group. After surgery, tramadol (1 mg kg^−1^) (Solarbio, Beijing, China) was administered intramuscularly for analgesia. Rats were placed on a warming pad until full recovery. The 0.3 W/cm^2^ + CPLLA group received daily ultrasound stimulation until the end of the experiment (parameters: intensity 0.3 W/cm^2^, frequency 1.0 MHz, duty cycle 50%, applied for 2 min per day).

#### 4.5.2 Serum Chemistry

Following the collection of rat blood samples, the samples must be left to stand at room temperature for a period of 30 min. Centrifugation at 3000 rpm for 10 min is required to achieve the separation of serum. The serum obtained should then be stored at −80°C in order to be analyzed at a later point. The Specialised Medical Centre of the People's Armed Police Force will conduct clinical serum testing to assess hepatic and renal function, including the following parameters: alkaline phosphatase (ALP), albumin (ALB), globulin (GLO), total protein (TP), alanine aminotransferase (ALT), aspartate aminotransferase (AST), creatinine (Cr), and urea (Urea).

#### 4.5.3 HE Staining

Initially, cardiac, hepatic, splenic and pulmonary tissue samples were fixed in 4% paraformaldehyde for a period of 24 h. Subsequently, paraffin embedding was performed, yielding 5‐micrometre‐thick sections. Following dewaxing and rehydration, sections underwent hematoxylin staining for 5 min, followed by counterstaining with blue and eosin staining for 2 min. Subsequently, the specimens underwent a process of graded ethanol dehydration and xylene clearing, followed by neutral resin mounting for examination. The staining pattern should manifest as blue nuclei, pink cytoplasm and collagen fibres, with clear and intact tissue architecture.

### 4.6 Neurological Function Assessment

#### 4.6.1 mNSS

The modified neurological severity score (mNSS) was administered on days 1, 3, 7, 14, 21, and 28 post‐TBI in order to evaluate neurological recovery (n = 10 per group). The mNSS employs an 18‐point scale, wherein higher scores indicate more severe neurological injury.

From days 22 to 28 post‐TBI surgery, the Morris water maze (MWM, DB001, Zhenghua, Anhui, China) was administered in order to evaluate cognitive function (n = 10 per group). Prior to the commencement of the training, the platform was situated in the third quadrant, with the rats positioned in such a manner that their dorsal surfaces were oriented toward the wall within the water tank. The duration required for the rat to jump into the water and ascend the platform was termed the escape latency. The training programme was considered to have been successful if the rat was able to locate the platform and remained there for a minimum of 3 s. In the event that the rat failed to locate the concealed platform within the stipulated 90‐s timeframe, the training was deemed unsuccessful. Thereafter, the rat was guided to the platform location, and its escape latency was documented as 90 s. Each rat was subjected to one training session per day in each of the four quadrants. On the 28th day post‐TBI, the platform was removed, and the rats were subjected to a spatial search test to assess their cognitive function. The escape latency, number of platform crossings, and duration spent in the target quadrant were recorded and analyzed.

#### 4.6.2 Gs

Grip strength (GS) was measured in rats (n = 10) at 1, 3, 7, 14, and 28 days post‐injury using a digital push‐pull dynamometer (SH‐III series, Nanjing Suchai Measurement Instrument Co., Ltd.). Peak grip strength values were recorded for comparison. During testing, the contralateral forepaw of the brain‐injured rat was placed on a wire mesh grid. The tail was then gently pulled backward to induce retraction until the forepaw released the grid. Each rat underwent three consecutive trials, with a 10‐min rest interval between trials. The highest recorded value among the three trials was defined as the maximum daily grip strength for each animal.

### 4.7 Electrophysiological Assessment

#### 4.7.1 Mep

Motor evoked potentials (MEPs) were recorded in rats (n = 10) at 1, 3, 7, 14, and 28 days post‐injury using the Pclab series biomedical signal acquisition system (Beijing Weixin Sida Technology Co., Ltd.). Stimulation and recording were performed using stainless steel needle electrodes (diameter 0.25 mm, length 13 mm). The stimulating electrodes were placed over the motor cortex, while the recording electrode was inserted directly into the mid‐belly of the gastrocnemius muscle in the hind limb. A ground electrode was attached to the metal experimental platform. Stimulation was delivered as a single pulse with a width of 3.0 ms, an inter‐electrode spacing of 4.0 mm, and a voltage of 4.0 V. The recording electrode had a sampling frequency of 20 kHz and an inter‐electrode spacing of 4.0 mm. Signal amplitudes were automatically identified by the acquisition system.

#### 4.7.2 Immunofluorescence, Nissl, and Luxol Fast Blue (LFB) Staining

On day 28 post‐implantation, rats were euthanized under deep anesthesia (n = 3 per condition). This was followed by transcardial perfusion with saline and fixation with 4% paraformaldehyde for 30 min. Brain tissue from the injury site, including areas approximately 5 mm surrounding the lesion, was collected. The harvested tissues were embedded in paraffin and sectioned into 5 µm slices using a microtome. For immunofluorescence staining, sections were deparaffinized and subjected to antigen retrieval. Subsequently, the sections were incubated overnight at 4 °C with the following primary antibodies: mouse anti‐Synaptophysin (MA1‐213, Invitrogen, 1:200), mouse anti‐Neurofilament (ab7794, Abcam, 1:200), rabbit anti‐Map2 (17490‐1‐AP, Proteintech, 1:300), rabbit anti‐MBP (10458‐1‐AP, Proteintech, 1:300), mouse anti‐GAP43 (ab315198, Abcam, 1:200), rabbit anti‐PSD95 (51‐6900, Invitrogen, 1:200), mouse anti‐Iba‐1 (ab283319, Abcam, 1:200), rabbit anti‐iNOS (18985‐1‐AP, Proteintech, 1:200), and chicken anti‐Arg‐1 (PA5‐143623, Invitrogen, 1:400). After three washes with PBS, the sections were incubated for 1 hour at room temperature with the following secondary antibodies: goat anti‐mouse IgG H&L (Alexa Fluor 488, ab150117, Abcam, 1:200), goat anti‐rabbit IgG H&L (Alexa Fluor 594, ab150084, Abcam, 1:200), and goat anti‐chicken IgY H&L (Alexa Fluor 555, ab150174, Abcam, 1:200). Nuclei were counterstained with DAPI. For Nissl staining, deparaffinized and rehydrated sections were rinsed with distilled water and stained with 0.5% cresyl violet solution for 3 min. The sections were then dehydrated, cleared in xylene, and observed under an inverted microscope. For Luxol fast blue (LFB) staining, sections were deparaffinized, rehydrated, and rinsed with distilled water before being stained in pre‐warmed LFB solution at 65°C for 4 h. After cooling, the sections were rinsed under running water until colorless, differentiated in lithium carbonate solution until myelin was distinctly blue and the background nearly colorless (monitored microscopically), and rinsed again to stop the reaction. Following dehydration in absolute ethanol, the sections were briefly counterstained with eosin, routinely dehydrated, cleared, and finally mounted with neutral balsam for examination. All stained sections were scanned using a slide scanning system (3DHISTECH, Budapest, Hungary).

#### 4.7.3 Mri

Imaging experiments were conducted on a 4.7 T animal MRI system (Bruker Biospin, USA) equipped with an active decoupled cross‐coil configuration. This comprised a 70 mm body coil for electrical transmission and a 25 mm surface coil for signal reception. Magnetic resonance imaging (MRI) data were collected on day 28 following TBI, encompassing T1, T2, and diffusion‐weighted imaging (DWI) sequences. Initially, cross‐sectional T1 images were acquired utilizing the following parameters: repetition time (TR) of 10.7 milliseconds, echo time (TE) of 3.8 milliseconds, echo chain number (EC) of 1, slice thickness of 0.8 millimetres, slice spacing of 0.4 millimetres, and receive bandwidth (BW) of 20.8 kilohertz. The acquisition parameters for T2‐weighted images were as follows: repetition time (TR) 3000 milliseconds, echo time (TE) 66.6 milliseconds, echo sequence (EC) 1, slice thickness 1.5 millimetres, slice gap 0 millimetres, receive bandwidth (BW) 25 kilohertz. The acquisition parameters for the DWI images were as follows: The SE/EPI sequence was utilised, with a repetition time (TR) of 2306 milliseconds, an echo time (TE) of 64.7 milliseconds, an echo chain number (EC) of 1, a slice thickness of 1.5 millimetres, a slice spacing of 0 millimetres, and a receive bandwidth (BW) of 250 kilohertz.

#### 4.7.4 Laser Speckle

This experiment used laser speckle contrast imaging (LSCI) technology to monitor cerebral hemodynamics in the rat cortex in vivo, non‐invasively and in real time, using a blood flow imaging system (MoorFLPI‐2, Germany). The rats were anesthetized with isoflurane and fixed in a stereotaxic apparatus with a thin skull window prepared to expose the sensorimotor cortex. The parameters of the 785 nm laser source and CCD camera were then adjusted to ensure that the sampled signal remained within the linear response range. After acquiring baseline speckle images continuously, their dynamic changes were recorded. The effects of traumatic brain injury (TBI) and subsequent radiofrequency ablation on microcirculation were evaluated by analyzing the spatiotemporal dynamics of blood flow within specific regions of interest (ROIs).

#### 4.7.5 RNA Sequencing and Analysis

In animal experiments, brain tissues were harvested from anesthetized mice on day 28 post‐TBI following established protocols. Cortical tissue within a 2 mm radius from the lesion core was defined as the peri‑lesional area and used for RNA‑seq analysis. Corresponding cortical regions from sham‑operated rats were collected as controls. In cell experiments, samples from the normal differentiation group and the 0.3 W/cm^2^ + CPLLA group were collected at day 14. All samples were snap‐frozen in liquid nitrogen and stored at −80°C until RNA extraction. Total RNA was extracted using TRIzol reagent. Sequencing libraries were constructed with the Illumina TruSeq Stranded mRNA Library Preparation Kit and sequenced on an Illumina NovaSeq 6000 platform. Raw sequencing reads were aligned to the mouse reference genome using HISAT2, followed by removal of rRNA transcripts. Gene expression levels were estimated using StringTie, which generated raw read counts and FPKM values. The FPKM values were used to plot sample correlation diagrams to assess global expression profiles. Differential expression analysis was performed based on raw counts using the DESeq2 package. Genes with |log_2_ (fold change)| ≥ 1 and an adjusted *p* < 0.05 were considered statistically significant. Volcano plots were generated to visualize the distribution of differentially expressed genes. Hierarchical clustering analysis was conducted using log_2_‐transformed counts per million (CPM) values, and heatmaps were plotted using the LC Bio Cloud Platform.

### 4.8 Statistical Methods

ImageJ image processing software was employed to quantitatively analyze protein expression levels and fluorescence intensity data from sample images. Experimental data are presented as mean ± standard deviation. Statistical analysis was performed using GraphPad Prism 8.0 software, employing unpaired *t*‐tests, one‐way analysis of variance (ANOVA) with Tukey's multiple comparison test, or two‐way ANOVA with Bonferroni multiple comparison test. Significant differences were determined using the following criteria: ^*^ *p* < 0.05, ^**^ *p* < 0.01, ^***^ *p* < 0.001, and ^****^ *p* < 0.0001.

## Conflicts of Interest

The authors declare no conflicts of interest.

## Supporting information




**Supporting File 1**: advs74613‐sup‐0001‐SuppMat.docx.

**Supporting File 2**: advs74613‐sup‐0002‐Movie S2‐S5.zip.

## Data Availability

The data that support the findings of this study are available from the corresponding author upon reasonable request.
