# Supplementary material for: Ultrasound Activated Piezoelectric Dural Patches to Drive Endogenous Neural Stem Cell–Mediated Repair Traumatic Brain Injury
Source: Adv Sci (Weinh). 2026 Mar 5;13(27):e24326. doi: 10.1002/advs.202524326 (PMC13170245; doi:10.1002/advs.202524326)
Supplement: Supplementary file 1 — Supporting File 1: advs74613‐sup‐0001‐SuppMat.docx. [file ADVS-13-e24326-s002.docx]

Supporting Information

## **Ultrasound Activated Piezoelectric Dural Patches to Drive Endogenous Neural Stem Cell–Mediated Repair Traumatic Brain Injury**

*Pengbo Zhou^#^, Qingyuan Wu^#^, Yang Wu , Runzhe Huang, Wei Li, Hanjie Niu, Hongtao Sun^*^ , Huiyu Liu^*^*

**Supplementary Methods**

**Simulated calculation:**

Periodic DFT calculations were carried out using the CP2K code.^[1]^ All calculations employed a mixed Gaussian and planewave basis sets. Core electrons were represented with norm-conserving Goedecker-Teter-Hutter pseudopotentials,^[2–4]^ and the valence electron wavefunction was expanded in a double-zeta basis set with polarization functions along with an auxiliary plane wave basis set with an energy cutoff of 450 Ry^[5]^. The generalized gradient approximation exchange-correlation functional of Perdew, Burke, and Enzerhof (PBE) was used.^[6]^ Test calculations showed that the total energy change of the reactive system was negligible (<0.01 eV) when the maximum force convergence criteria of 0.001 hartree/bohr was used. Each reaction state configuration was optimized with the Broyden-Fletcher-Goldfarb-Shanno (BGFS) algorithm with SCF convergence criteria of 1.0×10^-5^ au. To compensate the long-range van der Waals dispersion interaction between the adsorbate and the framework, the DFT-D3 scheme with an empirical damped potential term was added into the energies obtained from exchange-correlation functional in all calculations^[7]^. Transition states of elementary steps in the dehydration and etherification reaction routes were located using the climbing image nudged elastic band (CI-NEB) method with seven intermediate images along the reaction pathway between the initial and the final states^[8,9]^. Each identified transition state was further confirmed by the vibrational frequency analysis. The AIMD simulations were performed in the canonical ensemble (NVT) for PLLA/Al_2_O_3_ (110) systems at 378.15 K. The configuration of PLA adsorbed on Al_2_O_3_ (110) surface was adopted as the initial structure for AIMD simulations. The temperature of the AIMD simulated system was controlled using a Nosé thermostat. Production runs were 20 *ps* long and a time step of 1.0 fs, where the first 2 *ps* was used for equilibrium and the last 18 *ps* for statistics analysis.

The Gibbs free energy change for each elementary step was calculated at 378.15 K (105℃), defining as follows:

$$\Delta G= \Delta E_{DFT}+\Delta E_{ZPE}-T\Delta S$$

where $\Delta E$ is the difference of electronic energy calculated with CP2K, $\Delta E_{ZPE}$ is the difference of zero-point energy (ZEP), and $T\Delta S$ is the changed entropy value. $E_{ZPE}$and $TS$ were calculated using the following equations for each reaction intermediates,

$$E_{ZPE}= \frac{1}{2} \sum_{i} hv_{i}$$

$$TS= {\sum_{i} hv_{i}\left( \frac{1}{e^{\frac{hv_{i}}{k_{B}T}}} \right) - k}_{B}T\sum_{i} \ln\left( 1- e^{\frac{-hv_{i}}{k_{B}T}} \right)$$

where $h$, $v_{i}$, and $k_{B}$ are Planck’s constant, vibrational frequencies, and Boltzmann constant, respectively.

The adsorption Gibbs free energy between the adsorbate and the substrate can be calculated using the following equation:

$\Delta G_{ads}$ = $G_{adsorbate@\mathrm{substrate}}$ - $G_{\mathrm{substrate}}$ - $G_{adsorbate}$

Where, $G_{adsorbate@\mathrm{substrate}}$ and $G_{\mathrm{substrate}}$ represent the total Gibbs free energies of the substrate with and without the adsorption of adsorbate, respectively. $G_{adsorbate}$ is the total Gibbs free energy of the adsorbate. According to this equation, a negative adsorption energy corresponds to a stable adsorption structure.

The Al_2_O_3_ (110) surface was represented by a periodic (3 × 4) supercell with the lattice constants of 20.9891 × 20.5139 × 19.5639 Å^3^ and α = 90.0000°, β = 90.0000°, γ = 95.8434°. A vacuum height of 18 Å in the Z direction was inserted between the periodic Al_2_O_3_ (110) surface slabs to eliminate any unphysical interaction. The atoms in the bottom two layers were frozen while all other atoms in the surface slab were relaxed in all DFT calculations.

**Animal Care and Ethical Approval**

The husbandry and use of laboratory animals strictly adhered to the protocols approved by the Ethics Committee of the Chinese People's Armed Police Force Medical Centre (Ethics Approval Number: 2025-006). SPF-grade SD rats (supplied by Beijing SPF Biotechnology Co., Ltd., RRID: RGD 737805) aged 8 weeks and weighing 220–250 grams were employed in the study. The housing conditions maintained a 12-hour circadian rhythm, with ad libitum access to food and water, and fasting occurred only prior to anaesthesia.

**Supplementary Figures**


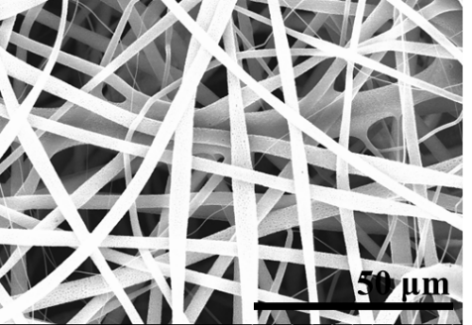


Figure S1. Representative morphology SEM of fibers electrospun at a collector speed of 300 rpm with dichloromethane as the solvent.


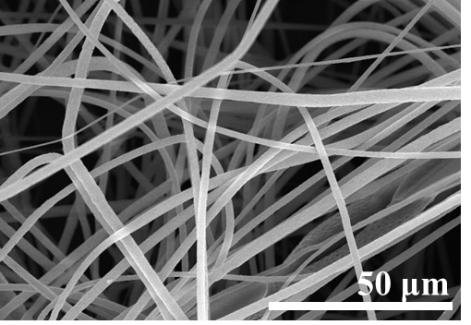


Figure S2. Representative morphology SEM of fibers electrospun at a collector speed of 300 rpm with hexafluoroisopropanol as the solvent.


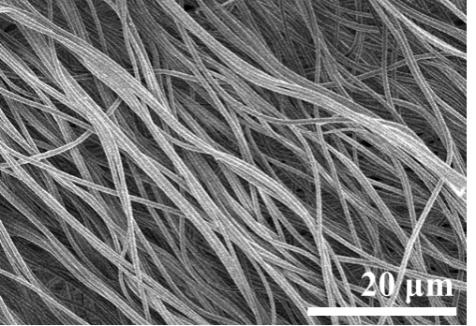


Figure S3. Representative morphology SEM of fibers electrospun at a collector speed of 1600 rpm with hexafluoroisopropanol as the solvent.


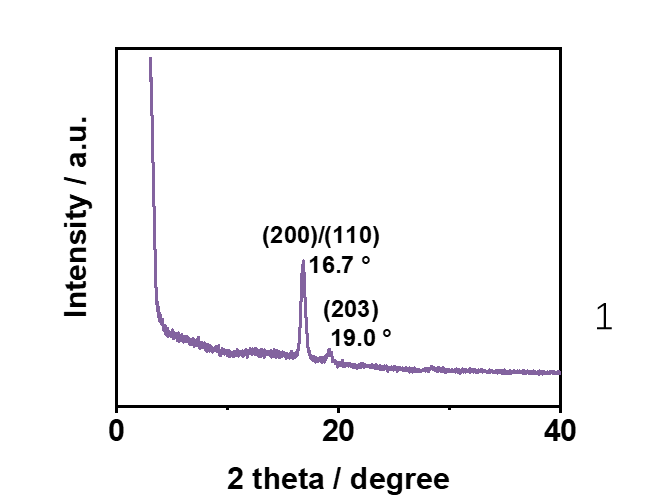


Figure S4. XRD pattern of the PLLA after conventional thermal treatment, confirming the formation of the α-crystal polymorph.


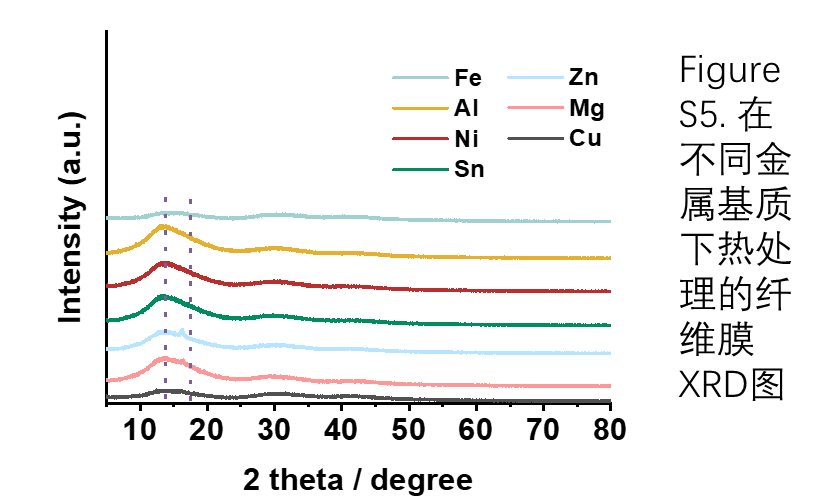


Figure S5. XRD patterns of PLLA membranes crystallized on various metal substrates, compared to the control sample.


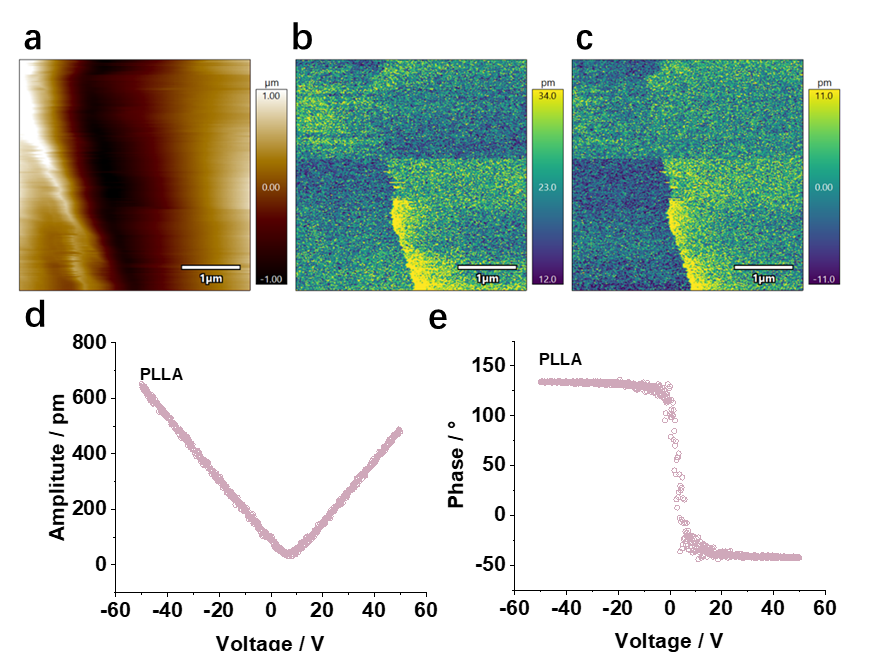


Figure S6. PFM analysis of the piezoelectric response. a,b, the amplitude curve (a) and phase curve (b) of PLLA. a-e, surface topography (a), amplitude plot (b), phase diagram (c), amplitude curve (d), and phase curve (e) of CPLLA.

*
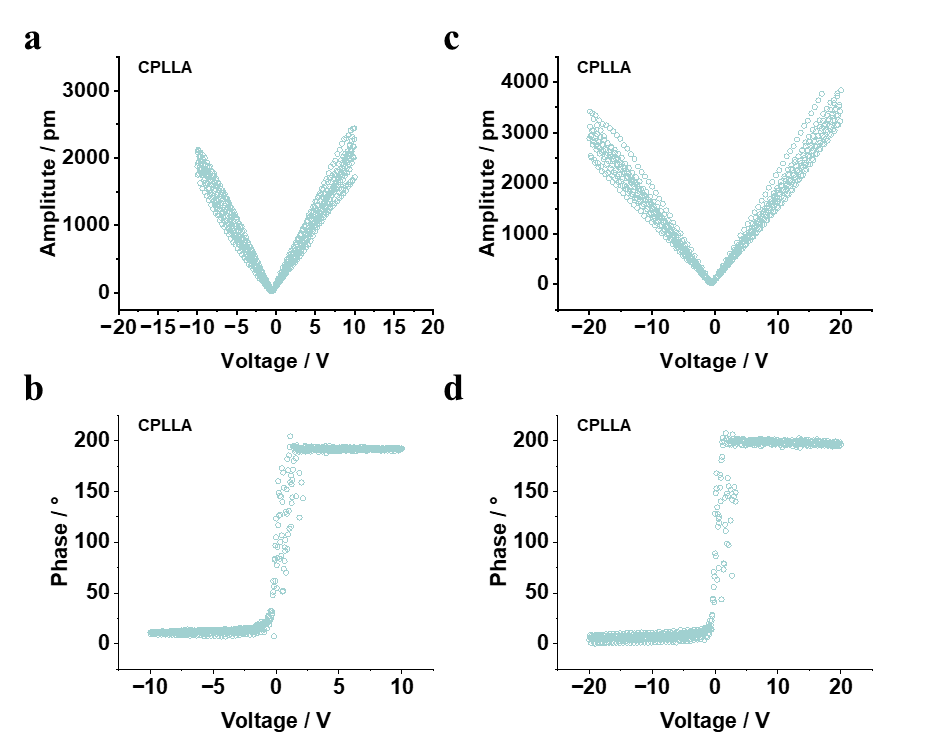
*

Figure S7. PFM analysis of the piezoelectric response for CPLLA. a) The amplitude curve and b) phase curve with 10 V bias voltage. c) The amplitude curve and d) phase curve with 20 V bias voltage.


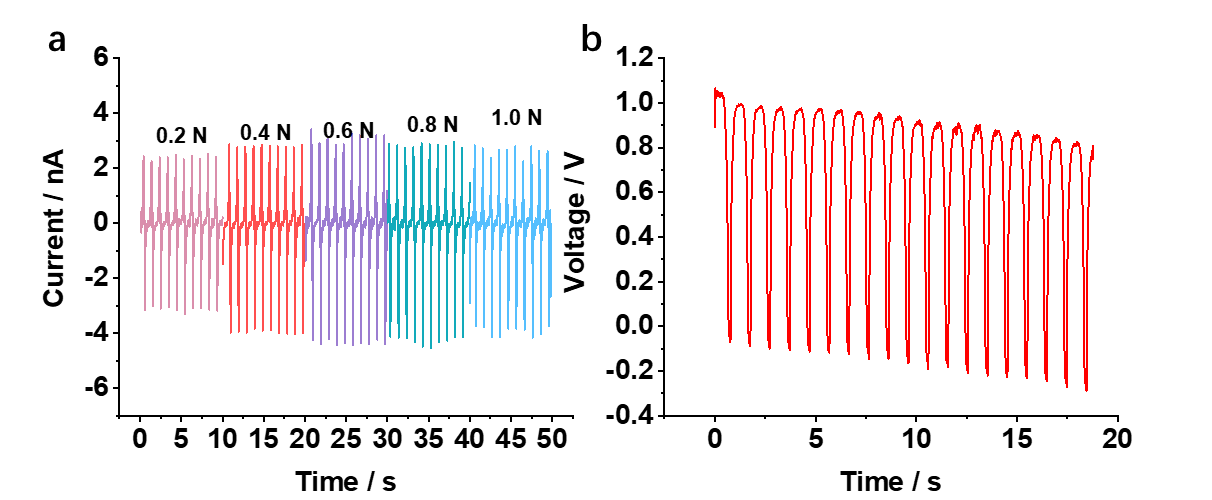


Figure S8. Current output a) and voltage output b) of CPLLA under different force.


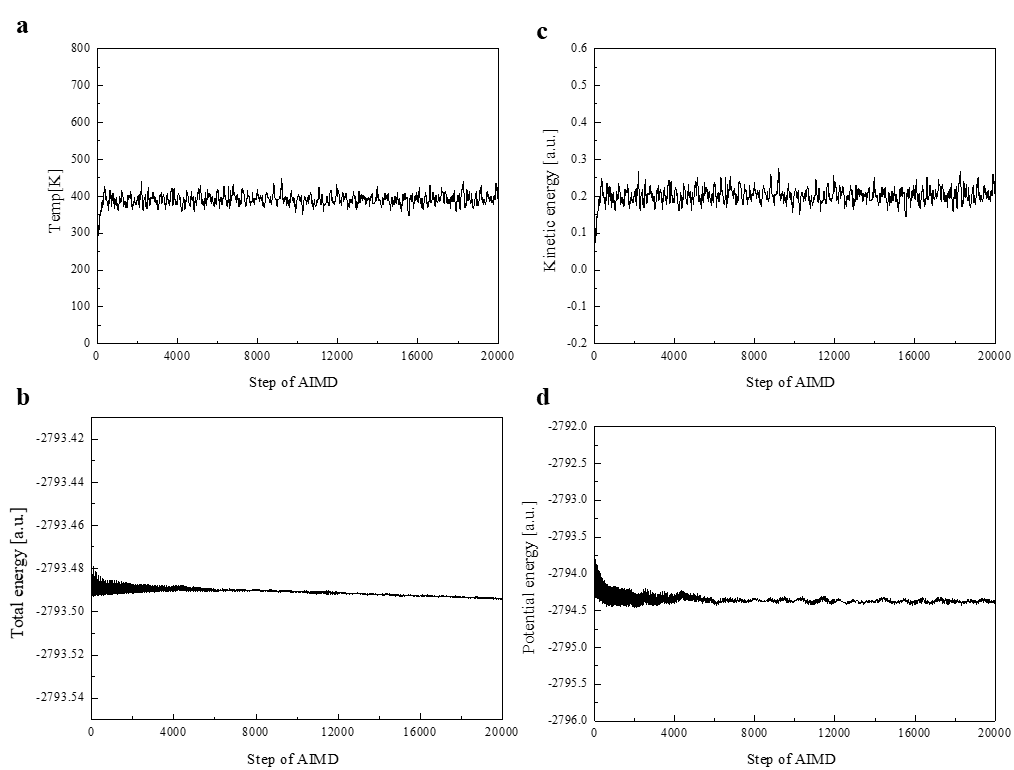


Figure S9. a) Curve of thermodynamic temperature as a function of step size of AIMD of Al_2_O_3_ (110) system. b) Curve of total energy (the sum of potential energy and kinetic energy) as a function of step size of AIMD of Al_2_O_3_ (110) system. c) Curve of kinetic energy as a function of step size of AIMD of Al_2_O_3_ (110) system. d) Curve of potential energy as a function of step size of AIMD of Al_2_O_3_ (110) system.

*
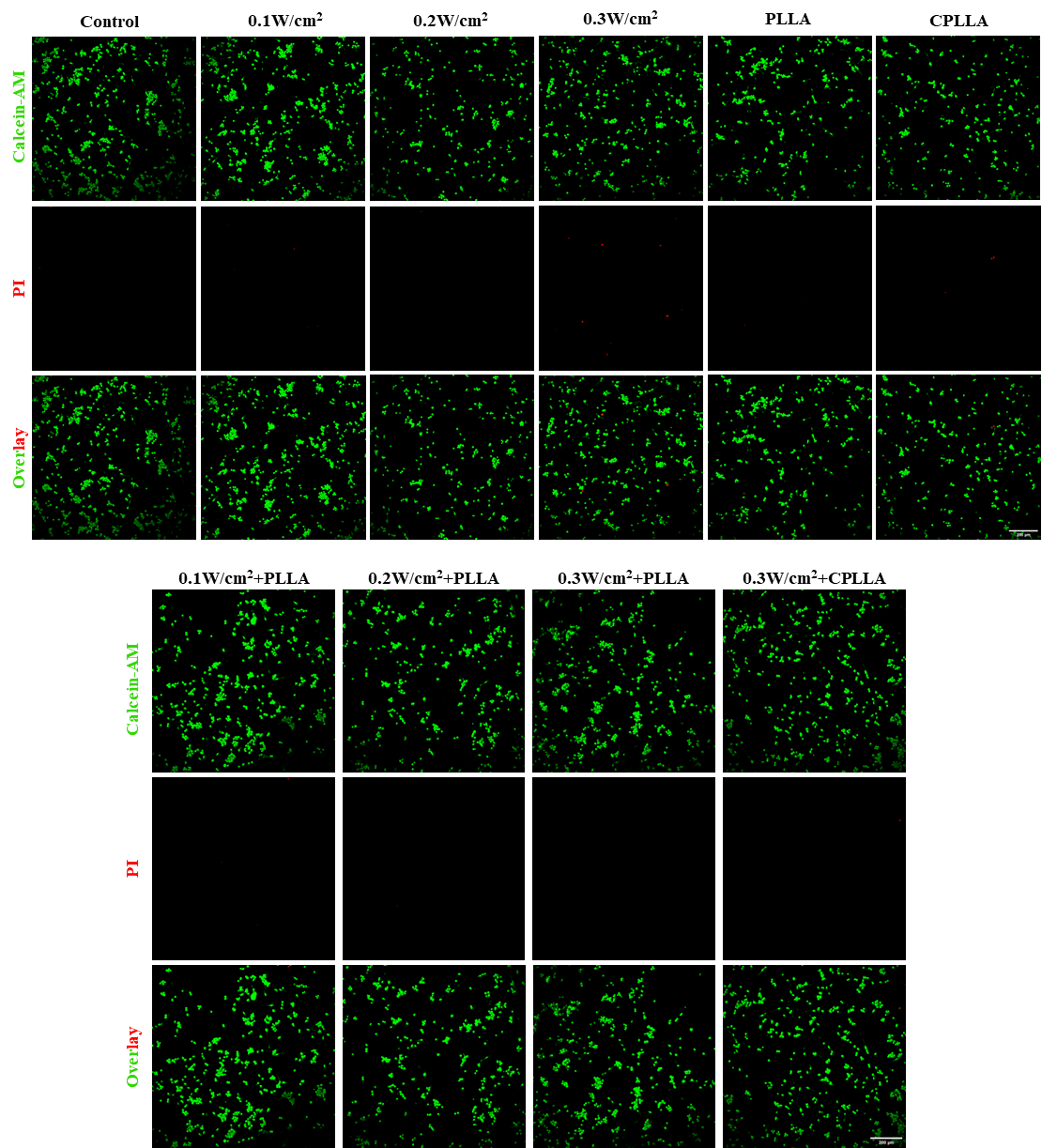
*

Figure S10. Live/dead cell staining images of BV2 cells after 24 hours under different conditions.
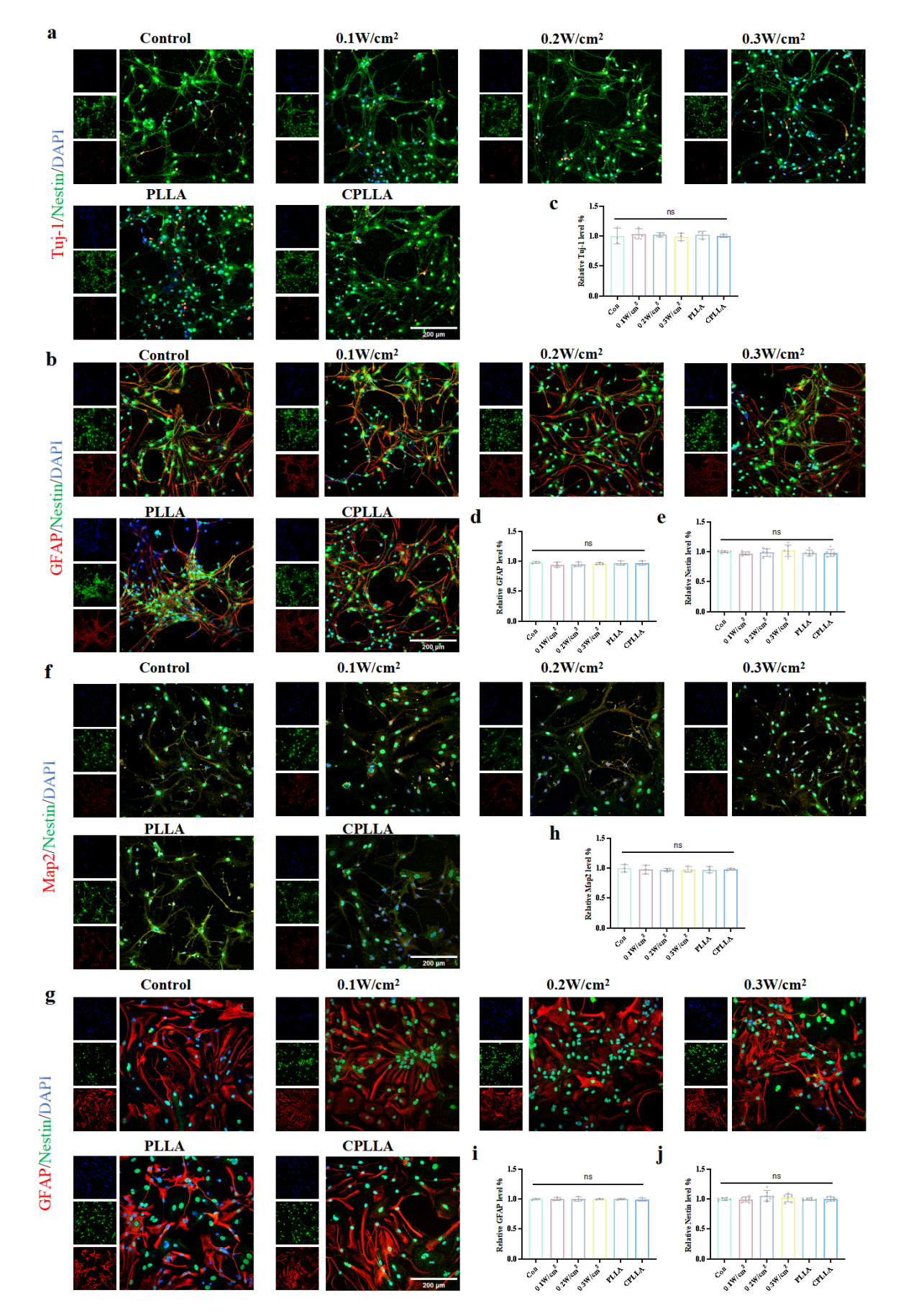


Figure S11. a-b) Fluorescent images of NSCs differentiated for 7 days following exposure to individual sound intensities or individual PLLA and CPLLA. Fluorescent expression levels of c) Tuj-1, d) GFAP, and e) Nestin at day 7 of neural stem cell differentiation; f-g) Fluorescent images of NSCs differentiated for 14 days following exposure to individual sound intensities or individual PLLA and CPLLA. Fluorescent expression levels of h)Map2, i) GFAP, and j) Nestin at day 14 of neural stem cell differentiation. , n = 3 independent biological replicates. Statistical analysis was performed using one-way ANOVA followed by multiple comparisons (NS: not significant; *P < 0.05, **P < 0.01, ***P < 0.001).


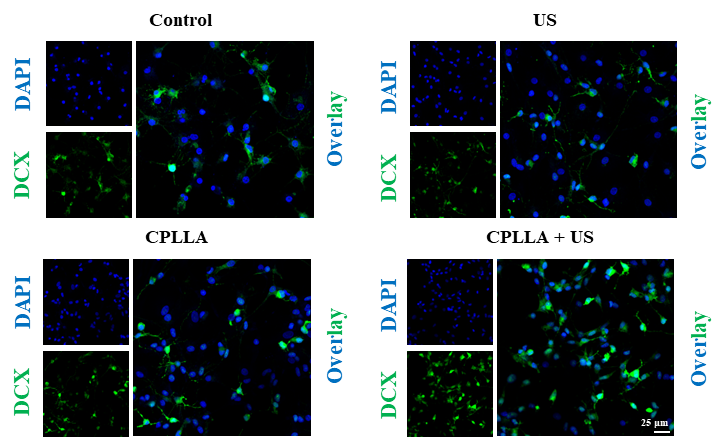


Figure S12. Representative immunofluorescence images of neural stem cells (NSCs) stained for DCX (green) and DAPI (blue) after treatment under indicated conditions.


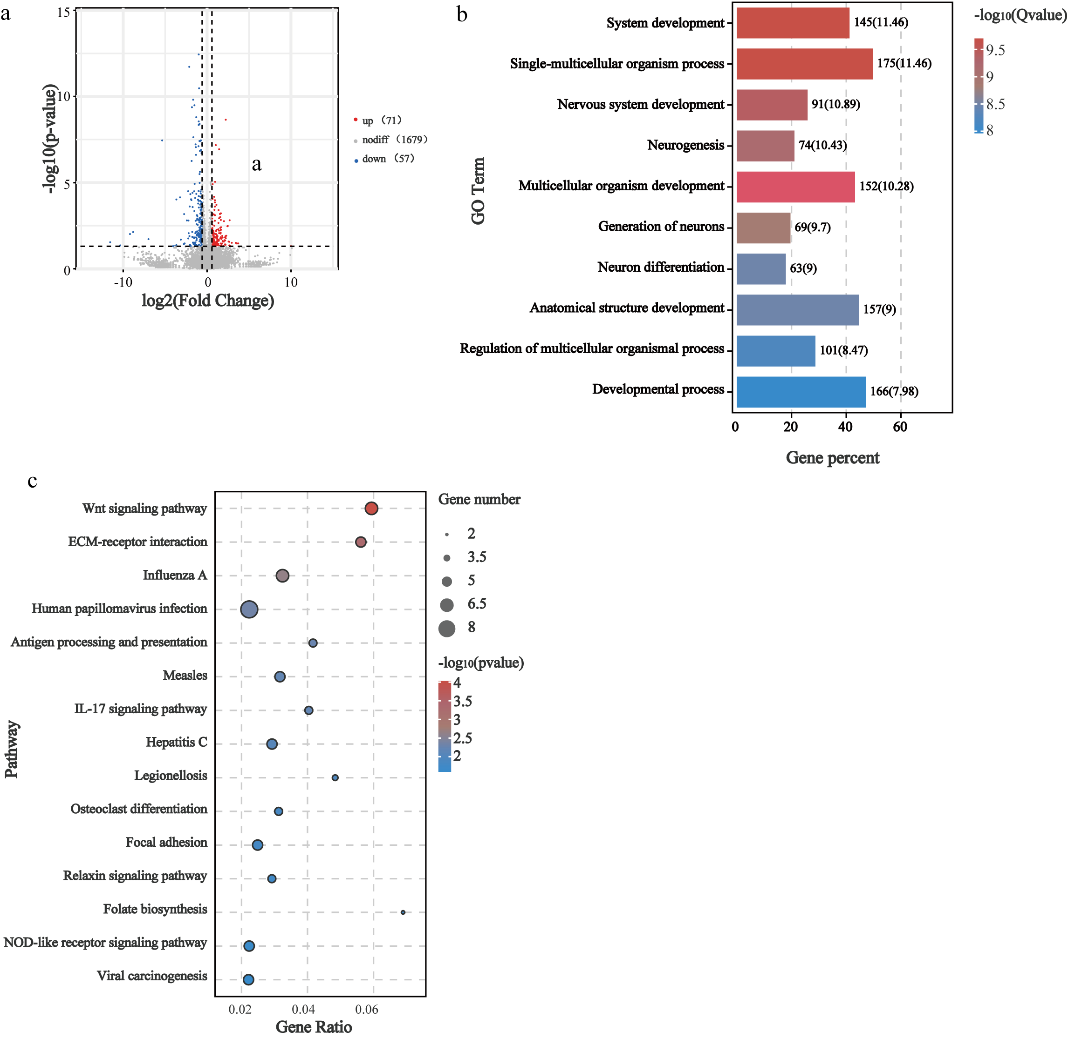


Figure S13. a) Volcano plot of differentially expressed genes (normal differentiation group vs. 0.3 W/cm² + CPLLA group, n=3). Red dots represent up-regulated genes; blue dots represent down-regulated genes. b) Gene Ontology (GO) analysis illustrating the biological significance of the differentially expressed genes between the two groups. c) KEGG pathway enrichment analysis of differentially expressed genes across the three groups.


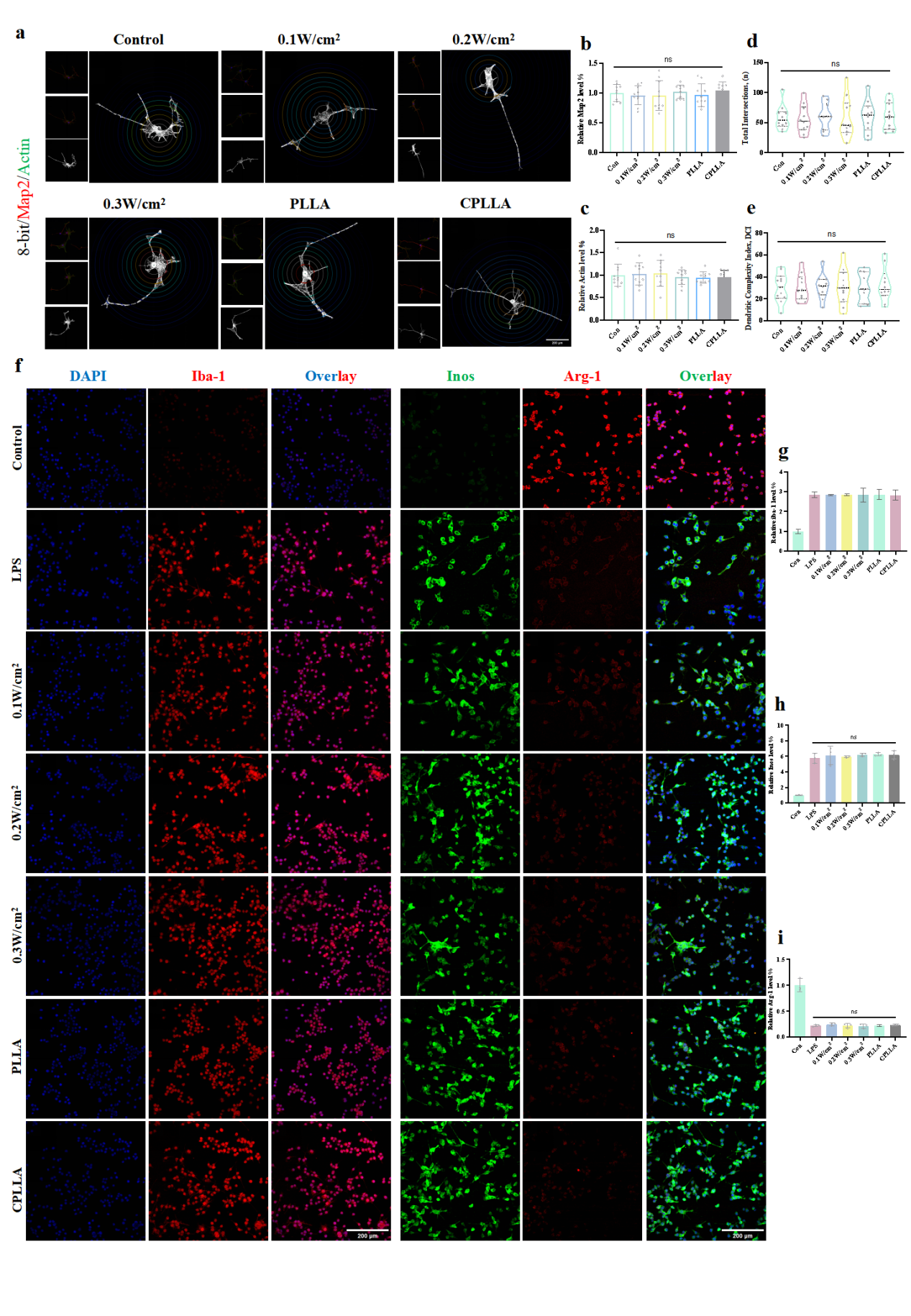


Figure S14 a) Sholl analysis of fluorescence images showing differentiation after 14 days of treatment with individual sound intensities or individual PLLA and CPLLA on NSCs. After 14 days under different conditions. b) Neuronal Map2 fluorescent expression, c) neuronal actin fluorescent expression, d) total neuronal crossings, and e) neuronal complexity index, n = 10 independent biological replicates. f) Fluorescence images showing activation and polarisation direction following LPS stimulation of BV2 cells treated with individual sound intensities or individual PLLA and CPLLA. Expression levels of h) inositol and i) Arg-1 protein blots in LPS-stimulated BV2 cells under different conditions, n = 3 independent biological replicates. Statistical analysis was performed using one-way ANOVA followed by multiple comparisons (NS: not significant; *P < 0.05, **P < 0.01, ***P < 0.001).


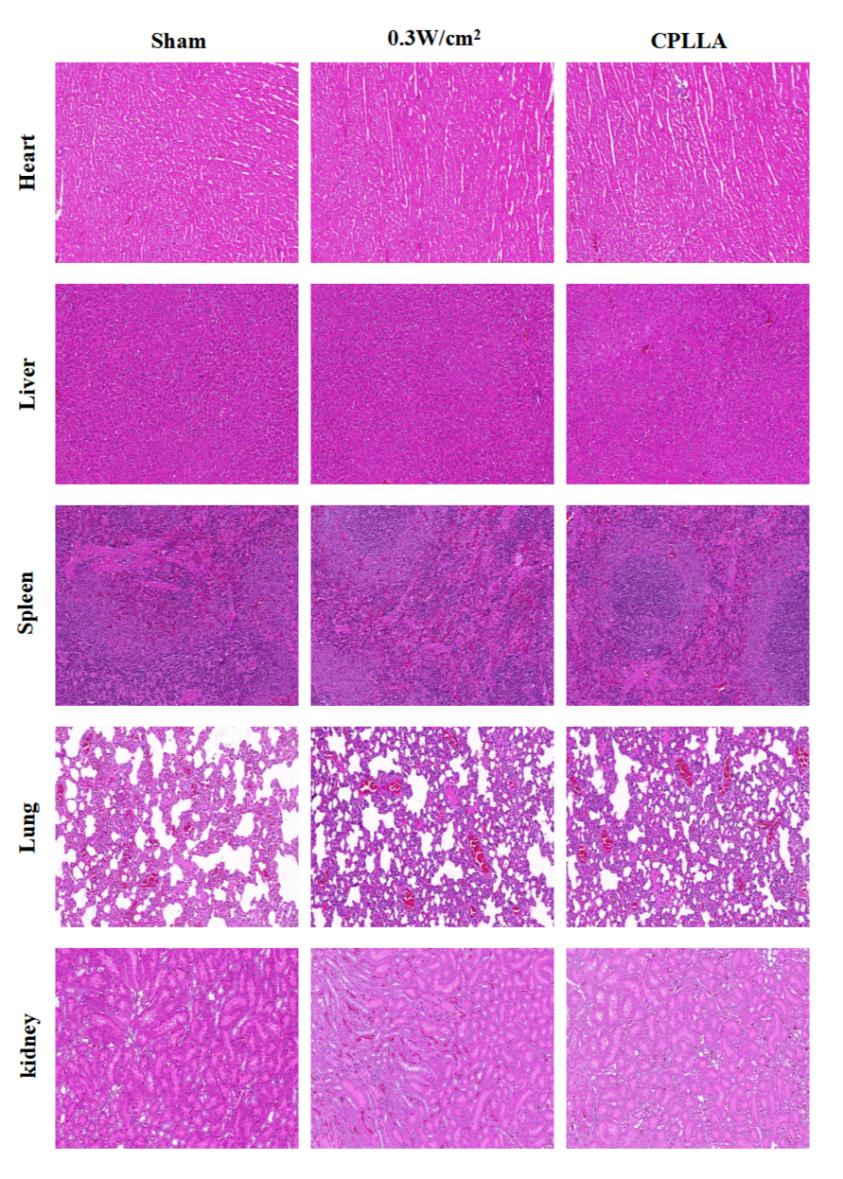


Figure S15. In vivo biocompatibility of ultrasound (0.3 W/cm²), CPLLA alone, and their combination (0.3 W/cm² + CPLLA) in rats.


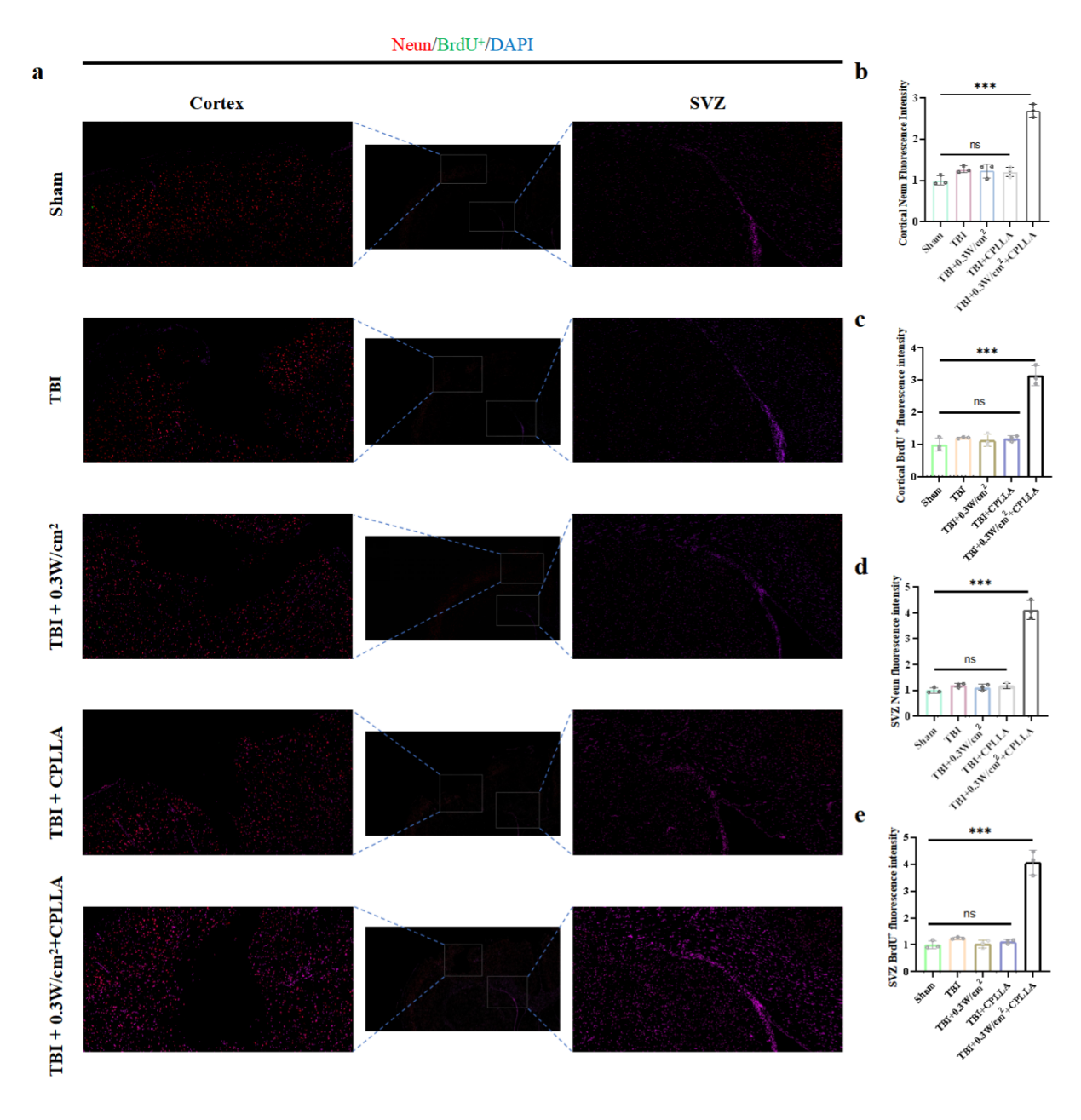


Figure S16. a) Representative immunofluorescence images showing NeuN and BrdU^+^ staining in brain tissue sections 28 days post-TBI in rats. Quantification of NeuN^+^ and BrdU^+^ cells in the cerebral cortex and subventricular zone (SVZ) (n=3). b) NeuN cell counts in the cortex. c) BrdU^+^ cell counts in the cortex. d) NeuN cell counts in the SVZ. e) BrdU^+^ cell counts in the SVZ. Statistical analysis was performed using one-way ANOVA followed by multiple comparisons (NS: not significant; *P < 0.05, **P < 0.01, ***P < 0.001).


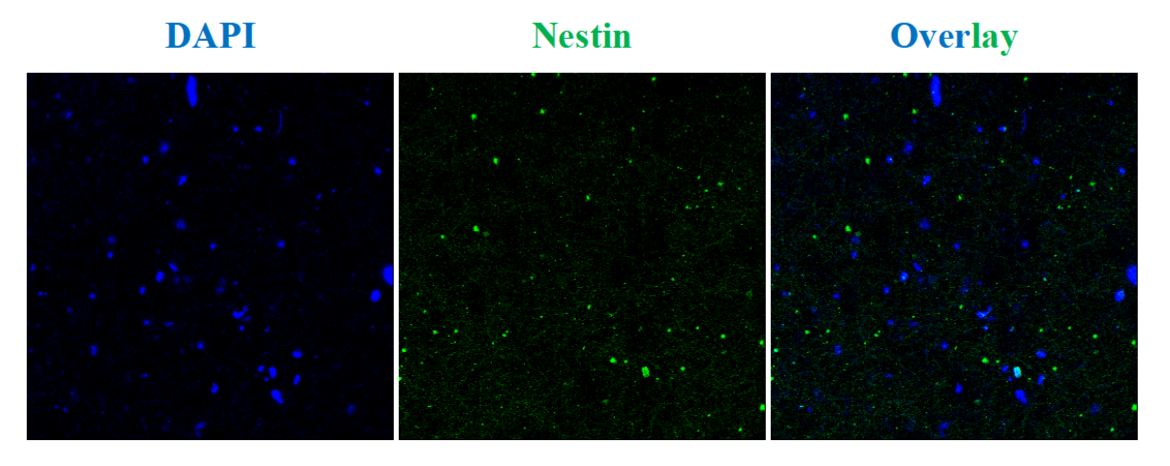


Figure S17. The neural stem cells on the materials that were implanted into the brains of traumatic brain injury rats after 28 days.

**Supplementary Tables**

Table S1 The piezoelectricity of CPLLA compared with other PLLA.

| PLLA^[10]^ | heat stretching | d14 = 9.57 pc/N |
| --- | --- | --- |
| PLLA^[11]^ | heat stretching | d14 = 10 pc/N |
| PLLA^[12]^ | heat stretching | d14 = 11 pc/N |
| PLLA^[13]^ | heat stretching | d14 = 13.1 pc/N |
| PLLA^[14]^ | electrospinning | d14 = 19 pc/N |
| PLLA^[15]^ | electrospinning | d33 = 0-30 pc/N |
| PLLA^[16]^ | electrospinning | d33 = 20 pc/N |
| PLLA/VB_2_^[17]^ | solution coating | d14 = 10 pc/N |
| PLLA/ZA^[18]^ | electrospinning | d14 = 8.02 pc/N |
| PLLA/PEG/BT^[19]^ | electrospinning | d14 = 10 pc/N |
| PLLA^[20]^ | electrospinning | d14 = 6-12 pc/N |
| PLLA^[21]^ | electrospinning | d33= 0.078 fc/N |
| PLLA^[22]^ | electrospinning | d14 = 10 pc/N |

Table S2 Primer sequences of each gene.

| **Target** | **Forward** | **Reverse** |
| --- | --- | --- |
| SCN8A | GCAAGCTCAAGAAACCACCC | CCGTAGATGAAAGGCAAACTCT |
| SCN2A | GCTAAGAGACCCAAACAGGAAC | GAATCGAGAGATTGCTTTCCCTT |
| KCNC1 | AGAGATTGGCACTCAGTGACT | TTGTTCACGATGGGGTTGAAG |
| KCNMA1 | CGACAAACAGAATGCAACAAGG | ACTCATGGGCTTGATTTGAATGT |
| IL-10 | GCTCTTACTGACTGGCATGAG | CGCAGCTCTAGGAGCATGTG |
| Arg-1 | CTCCAAGCCAAAGTCCTTAGAG | AGGAGCTGTCATTAGGGACATC |
| IL-1β | AGCAGCTTTCGACAGTGAGG | CTCCACGGGCAAGACATAGG |
| GAPDH | AGACAGCCGCATCTTCTTGT | CTTGCCGTGGGTAGAGTCAT |

**Reference**

[1] J. VandeVondele, M. Krack, F. Mohamed, M. Parrinello, T. Chassaing, J. Hutter, *Computer Physics Communications* **2005**, *167*, 103.

[2] S. Goedecker, M. Teter, J. Hutter, *Phys. Rev. B* **1996**, *54*, 1703.

[3] C. Hartwigsen, S. Goedecker, J. Hutter, *Phys. Rev. B* **1998**, *58*, 3641.

[4] M. Krack, M. Parrinello, *Phys. Chem. Chem. Phys.* **2000**, *2*, 2105.

[5] Thom. H. Dunning, P. J. Hay, in *Methods of Electronic Structure Theory* (Ed.: H. F. Schaefer), Springer US, Boston, MA, **1977**, pp. 1–27.

[6] J. P. Perdew, K. Burke, M. Ernzerhof, *Phys. Rev. Lett.* **1996**, *77*, 3865.

[7] S. Grimme, J. Antony, S. Ehrlich, H. Krieg, *The Journal of Chemical Physics* **2010**, *132*, 154104.

[8] J. VandeVondele, J. Hutter, *The Journal of Chemical Physics* **2007**, *127*, 114105.

[9] G. Mills, H. Jónsson, G. K. Schenter, *Surface Science* **1995**, *324*, 305.

[10] C. Zhao, J. Zhang, Z. L. Wang, K. Ren, *Advanced Sustainable Systems* **2017**, *1*, 1700068.

[11] J. Tian, F. Jiang, Q. Zeng, M. PourhosseiniAsl, C. Han, K. Ren, *IEEE Sensors J.* **2023**, *23*, 6264.

[12] E. J. Curry, K. Ke, M. T. Chorsi, K. S. Wrobel, A. N. Miller, A. Patel, I. Kim, J. Feng, L. Yue, Q. Wu, C.-L. Kuo, K. W.-H. Lo, C. T. Laurencin, H. Ilies, P. K. Purohit, T. D. Nguyen, *Proc. Natl. Acad. Sci. U.S.A.* **2018**, *115*, 909.

[13] F. Jiang, Y. Shan, J. Tian, L. Xu, C. Li, F. Yu, X. Cui, C. Wang, Z. Li, K. Ren, *Adv Materials Inter* **2023**, *10*, 2202474.

[14] E. J. Curry, T. T. Le, R. Das, K. Ke, E. M. Santorella, D. Paul, M. T. Chorsi, K. T. M. Tran, J. Baroody, E. R. Borges, B. Ko, A. Golabchi, X. Xin, D. Rowe, L. Yue, J. Feng, M. D. Morales-Acosta, Q. Wu, I.-P. Chen, X. T. Cui, J. Pachter, T. D. Nguyen, *Proc. Natl. Acad. Sci. U.S.A.* **2020**, *117*, 214.

[15] S. Chen, X. Wang, D. Zhang, Z. Huang, Y. Xie, F. Chen, C. Liu, *Regenerative Biomaterials* **2025**, *12*, rbae150.

[16] A. Farahani, A. Zarei-Hanzaki, H. R. Abedi, I. Haririan, M. Akrami, Z. Aalipour, L. Tayebi, *Journal of Materials Research and Technology* **2021**, *15*, 6356.

[17] Q. Zhang, Q. Liu, W. Xue, Y. Xiang, X. Hu, *Polymers* **2024**, *16*, 1071.

[18] X. Cui, Y. Shan, J. Li, M. Xiao, Y. Xi, J. Ji, E. Wang, B. Zhang, L. Xu, M. Zhang, Z. Li, Y. Zhang, *Adv Funct Materials* **2024**, *34*, 2403759.

[19] Q. Liu, L. Liu, D. Fan, S. Xie, C. Wang, X. Gou, X. Li, *Applied Materials Today* **2024**, *37*, 102120.

[20] M. T. Chorsi, E. J. Curry, H. T. Chorsi, R. Das, J. Baroody, P. K. Purohit, H. Ilies, T. D. Nguyen, *Advanced Materials* **2019**, *31*, 1802084.

[21] A. Farahani, A. Zarei-Hanzaki, H. R. Abedi, I. Haririan, M. Akrami, Z. Aalipour, L. Tayebi, *Journal of Materials Research and Technology* **2021**, *15*, 6356.

[22] M. Smith, Y. Calahorra, Q. Jing, S. Kar-Narayan, *APL Materials* **2017**, *5*, 074105.
